# Spatial heterogeneity of tumor microenvironment influences the prognosis of clear cell renal cell carcinoma

**DOI:** 10.1186/s12967-023-04336-8

**Published:** 2023-07-20

**Authors:** Dawei Zhang, Yuanli Ni, Yongquan Wang, Juan Feng, Na Zhuang, Jiatao Li, Limei Liu, Wenhao Shen, Ji Zheng, Wei Zheng, Cheng Qian, Juanjuan Shan, Zhansong Zhou

**Affiliations:** 1 Department of Urology, The Southwest Hospital, Army Medical University, No. 30 Gaotanyan Street, Shapingba District, Chongqing, 400038 China; 2 Chongqing Key Laboratory of Translational Research for Cancer Metastasis and Individualized Treatment, Chongqing University Cancer Hospital, No. 181 Hanyu Road, Shapingba District, Chongqing, 400030 China; 3 Anesthesiology Department, The 80th Army Hospital of Chinese PLA, Weifang, 261021 Shandong China

**Keywords:** Clear cell renal cell carcinoma, Tumor microenvironment, Immune architecture, Cellular neighborhood, Survival

## Abstract

**Background:**

Clear cell renal cell carcinoma (ccRCC) is an immunologically and histologically diverse tumor. However, how the structural heterogeneity of tumor microenvironment (TME) affects cancer progression and treatment response remains unclear. Hence, we characterized the TME architectures of ccRCC tissues using imaging mass cytometry (IMC) and explored their associations with clinical outcome and therapeutic response.

**Methods:**

Using IMC, we profiled the TME landscape of ccRCC and paracancerous tissue by measuring 17 markers involved in tissue architecture, immune cell and immune activation. In the ccRCC tissue, we identified distinct immune architectures of ccRCC tissue based on the mix score and performed cellular neighborhood (CN) analysis to subdivide TME phenotypes. Moreover, we assessed the relationship between the different TME phenotypes and ccRCC patient survival, clinical features and treatment response.

**Results:**

We found that ccRCC tissues had higher levels of CD8^+^ T cells, CD163^−^ macrophages, Treg cells, endothelial cells, and fibroblasts than paracancerous tissues. Immune infiltrates in ccRCC tissues distinctly showed clustered and scattered patterns. Within the clustered pattern, we identified two subtypes with different clinical outcomes based on CN analysis. The TLS-like phenotype had cell communities resembling tertiary lymphoid structures, characterized by cell–cell interactions of CD8^+^ T cells-B cells and GZMB^+^CD8^+^ T cells-B cells, which exhibited anti-tumor features and favorable outcomes, while the Macrophage/T-clustered phenotype with macrophage- or T cell-dominated cell communities had a poor prognosis. Patients with scattered immune architecture could be further divided into scattered-CN-hot and scattered-CN-cold phenotypes based on the presence or absence of immune CNs, but both had a better prognosis than the macrophage/T-clustered phenotype. We further analyzed the relationship between the TME phenotypes and treatment response in five metastatic ccRCC patients treated with sunitinib, and found that all three responders were scattered-CN-hot phenotype while both non-responders were macrophage/T-clustered phenotype.

**Conclusion:**

Our study revealed the structural heterogeneity of TME in ccRCC and its impact on clinical outcome and personalized treatment. These findings highlight the potential of IMC and CN analysis for characterizing TME structural units in cancer research.

**Supplementary Information:**

The online version contains supplementary material available at 10.1186/s12967-023-04336-8.

## Introduction

Renal cell carcinoma (RCC) is among the top 10 cancers worldwide, of which the clear cell variant is one of the most common and aggressive subtypes [1–3]. Early ccRCC can be well managed by surgery. However, advanced cases usually lose the chance of radical resection due to metastasis and are refractory to conventional chemotherapy and radiotherapy, thus having a disappointing survival rate. Fortunately, the advent of targeted drugs and immunotherapy has brought promising prospects for the treatment of ccRCC [4]. However, a substantial proportion of ccRCC patients still respond poorly to the therapies, and the reason remains unclear [5–7].

The prognosis and therapeutic strategy of cancer patients are commonly based on the traditional AJCC-TNM staging. However, patients with the same clinical stage often have varying prognoses and immunotherapy responses. The tumor microenvironment (TME), especially the immune infiltrates, is crucial to the proliferation, differentiation and metastasis of cancer cells. Insight into TME or tumor immune microenvironment (TIME) may aid in personalized clinical management for cancer patients. The immunoscore has been proven to have advantages over the traditional ACJJ-TNM staging in colon cancer [8]. Furthermore, stratification systems regarding the location and density of immune infiltrates have also been proposed to guide therapeutic decisions [9]. However, these simplified stratification systems only targeted specific immune cell types, such as CD8^+^ T cells, and could not mimic the complex TIME. In recent years, high-throughput technologies such as single-cell sequencing, multicolor flow cytometry and mass cytometry have fully displayed their talents in deciphering the intricate TIME, which identified many immune cell subtypes associated with the survival, metastasis, recurrence and immunotherapy response of ccRCC [10–12]. To be noted, aside from specific immune cell subtypes, the spatial architecture and cell-to-cell interactions of infiltrating immune cells also affect the prognosis and treatment of cancer. For example, the tertiary lymphoid structure (TLS), which promotes anti-tumor antibody secretion, antigen presentation, better prognosis and immunotherapy response, has been found in various tumors, such as RCC, melanoma and ovarian cancer [13–16]. In fact, the immune structures other than TLS in cancer are quite diverse. Keren et al. [17] reported three immune-phenotypes in triple-negative breast cancer using multiplexed ion beam imaging by time of flight (MIBI-TOF), including cold, mix and compartment. Sheng J. et al. [18] revealed three intra-tumor regions (normal, fibrotic and cancerous regions) in hepatocellular carcinoma and identified a variety of cell neighborhoods (CNs) associated with patient prognosis. Another study on hepatocellular carcinoma reported that the cell-to-cell interactions in local functional areas could affect the immunotherapy response. The proximity of CD8^+^ T cells to CD4^+^ T cells, rather than arginase 1^high^ macrophages, promoted the response of immune checkpoint inhibitor (ICI) treatment [19]. These studies indicated that although a complex system, like an enormous genome encoding just a limited number of markers, the TME also has countable cell-to-cell interactions or CN patterns [20, 21]. Therefore, it is quite necessary and feasible to investigate the local cell communities to understand tumor progression and guide treatment.

ccRCC is an immunologically and histologically heterogeneous tumor. However, the impact of TME structural heterogeneity on ccRCC needs further exploration. In this study, we surveyed the TME of ccRCC by IMC and discovered two distinct immune architectures. Using CN analysis, we further identified four different phenotypes associated with the prognosis of ccRCC and response to sunitinib. These results deepened our understanding of the spatiotemporal heterogeneity of immune infiltrates in ccRCC and will facilitate personalized clinical management in the future.

## Methods and materials

### Patients and datasets

All samples used for phenotype identification were from the ccRCC tissue microarray (HKidE180Su03, Shanghai Outdo Biotech, Additional file 6: Table S1). We finally enrolled 75 qualified samples for analysis, excluding three non-ccRCC tissues, nine samples with insufficient tissues and three unsound scanning tissues. Through Imaging Mass Cytometry (IMC) scanning, we got 86 mcd files of regions of interest (ROI). The information of ccRCC patients and ROIs were supplied in Additional file 6: Tables S2 and S4. To explore what changes have taken place in the TME of ccRCC, we analyzed 13 cancerous and paired paracancerous tissues.

Five cancerous tissues from patients with metastatic ccRCC were collected at the Southwest Hospital, Chongqing, China (2019–2022). All patients received targeted therapy and/or immunotherapy after radical nephrectomy. Clinical information is supplied in Additional file 6: Table S6. Informed consent was received from each patient and the research design was approved by the Ethics Review Committee of Southwest Hospital, Army Medical University.

## Section staining procedure for IMC

### Section staining

Preheated the slide to 60 °C for 2 h and then immersed it into two m-xylene (X820562-500 ml, Macklin) vats separately to dewax for 10 min with loose lids. Next, we hydrated the slide in descending grades of ethanol (100%, 95%, 80% and 70%) for 5 min each. Preheated a centrifuge tube containing 40 ml diluted Tris–EDTA (10 × diluted to 1x, C1038, Solarbio) to 96 °C and incubated the hydrated slide with tissues for 30 min for antigen retrieval, leaving the lid loose to maintain internal and external pressure balance. After incubation, the centrifuge tube was cooled to 70 °C at room temperature (RT). The slide was then washed by ddH_2_O and DPBS (14190144, Thermo Fisher) for 10 min each. Used a PAP pen to circle all tissues on the slide and blocked with 3% Bovine Serum Albumin (BSA, SRE0096-50G, Sigma) in DPBS for 45 min at RT.

Prepared the antibody cocktail with 0.5%BSA in DPBS. The information of the antibodies and cell segmentation reagents are supplied in Additional file 6: Table S3. Removed the blocking solution, pipetted the antibody mix onto the slide with ccRCC tissues and incubated the slide in a hydration chamber overnight. Washed the slide in 0.2% Triton™X-100 (85111, Thermo Fisher) in DPBS twice for 8 min each. Washed the slide in DPBS twice for 8 min each. Incubated the slide with Ir-intercalator (201192, Fluidigm) in DPBS (1:400) for 30 min at RT. The slide was then washed in DPBS for 5 min and air-dried for 30 min at RT.

### IMC analysis

We used the Hyperion + ™ Imaging System (Fluidigm) to scan the tissues and obtained MathCaD (MCD) files containing multi-plexed images. In order to segment the image into single-cell data, we leveraged MCD Viewer software (V 1.0.5, Fluidigm) to convert MCD files to 16-bit multi TIFF files. Next, CellProfiler (V 4.1.3, Broad Institute) was used to generate cellmask files from the TIFF files. Finally, we placed the cellmask and TIFF files of each tissue in a separate folder and generated csv files using the histoCAT software (V 1.76) [22]. The files acquired above were subsequently used for cell clustering, cell type identification and cellular neighborhood analysis. The spatial distribution of different markers was visualized using MCD Viewer software. The phenograph method in histoCAT software was employed to cluster cells from 13 pairs of ccRCC and adjacent tissues, followed by tSNE dimensionality reduction. Wilcoxon's rank sum test was utilized to assess the statistical significance between different cell clusters of the 13 pairs of ccRCC and adjacent tissues.

## Dimensionality reduction, cluster identification and cell components analysis

The mean intensity and the pixel coordinates of the centroid of each cell were abstracted from the csv files. Next, we created a spatial image object for the cells in each ccRCC tissue using the seurat package (V 4.0.5) of R. After normalization with the SCTransform function, we merged all samples and select CD3, CD8a, CD20, CD4, CD68, CD31, Pan-keratin, Ecad, αSMA, and Vim as high-variant features for input into Principal Component Analysis (PCA). The findNeighbor function was utilized to define the edge weights between any two cells based on the shared overlap in their local neighborhoods, with the PCA set to 6 as input. Lastly, we employed the FindClusters function to perform cell clustering with a resolution of 0.6, and calculated uniform manifold approximation and projection (UMAP) embeddings using PCA with a value of 6.

We drew the mean marker intensity curves and set the knee points as their thresholds for each marker to determine the positive cells. The mean marker intensities in T cells, B cells, macrophages, tumor cells, epithelial cells, endothelial cells and fibroblasts, as well as the proportion and number of various cell types in each ccRCC tissues, were calculated with a self-designed R script. Heatmap illustrating the average expression of the markers was drafted by the pheatmap function in the pheatmap package (V 1.0.1) of R.

## Calculating mix score

We first defined T cells, B cells and macrophages as the T/B/M cells, and the rest of the cells as the other. A mixing score was established to quantify the degree of mixing between T/B/M cells and other cells. The mixing score for a patient was defined as the proportion of T/B/M cells touching other cells and was calculated as the number of T/B/M-other cell interactions divided by the number of T/B/M-T/B/M interactions in the neighbors’ matrix. The mixing_score_summary function in the SPIAT [23] (Spatial Image Analysis of Tissue, V 0.4) package of R was used to calculate the mix score of each patient (reference_marker = “T/B/M”, target_marker = “other”, radius = 30). If a patient had two or more ROI, we calculated a mean mix score as the final mix score.

Next, we abstracted the pixel coordinates of the centroid of each cell from the established “seurat spatial image object” and simulated the spatial conformation of each tissue and the spatial distribution of T/B/M using the ggplot function in the ggplot2 package (V 3.3.5) of R.

## Cellular neighborhood (CN) analysis

We used the identify_neighborhoods function (method =  “hierarchical”, min_neighhorhood_size = 10, radius = 10) in the SPIAT (V0.4) package of R software to identify CNs of each tissue. Cells of interest were those we identified and annotated in the study. While cells that could not be annotated were not analyzed. A R script was designed to analyze the proportion and number of various cell types in each CN. The CN with a proportion of a given cell type greater than 30% would be annotated as the cell type CN enriched. Additional file 6: Table S5 described the cell type annotation rules. We then applied the ggplot function in the ggplot2 package (V 3.3.5) of R to plot the spatial distribution of cells and CNs. Due to the abundance of T cells, B cells and macrophages infiltrating in the clustered group, we annotated the CN with a proportion of a given cell type greater than 20% as the cell type CN to detail and distinguish the immune interactions. For example, CD8^+^ T cell_CD163^-^ macrophage CN represents the co-enrichment of CD8^+^ T cells and CD163^-^ macrophage cells in that CN unit.

## Survival analysis

For a specific cell component, we first calculate its proportion in each tissue. Tissues with a proportion of the cells ≥ the third quartile (Q3) of all tissues were defined as the high infiltration group, and those ≤ the first quartile (Q1) of all tissues were allocated to the low infiltration group. The survfit function in the survival package (V 3.2-13) of R was used to model the overall survival (OS), and the survival curve was drafted using the ggsurvplot function.

The survival curve of the different phenotypes identified in this study was also plotted with the survival package (V 3.2-13) of R.

## Statistical analysis

Data were analyzed using the statistical package R (V 4.0.5). For a certain cell component, we used its quartiles to determine high (≥ Q3) and low groups (≤ Q1). The survival differences between the high and low cell type groups were analyzed by the R package 'survminer’, as were those between different phenotypes. The duration of survival was defined as the time from the date of diagnosis to the date of death or last known follow-up. The clinical characteristics of different phenotypes were compared by chi-square. We compared the proportions of different cell components in CNs or in total cells of each ccRCC tissue between different phenotypes. Distributions were compared by 2-sided Wilcoxon’s test rank sum test. P values < 0.05 were considered statistically significant.

## Results

### Spatial characteristics and differences between the ccRCC and paired paracancerous tissues

**Fig. 1:**
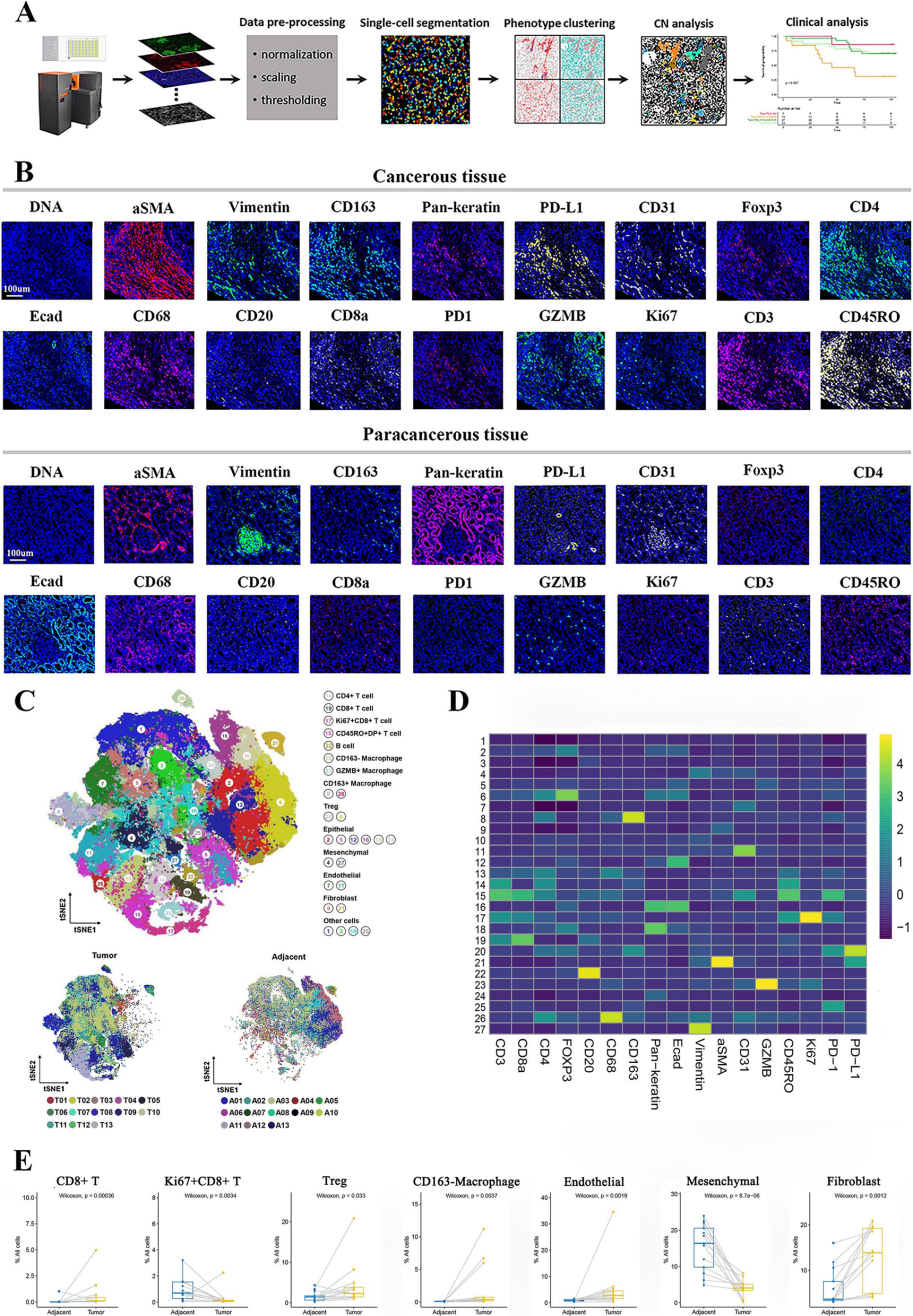
Spatial characteristics and differences between the ccRCC and paired paracancerous tissues. **A** Workflow of IMC data processing and analysis, including tissue preparation, antibody staining, image acquisition, single-cell segmentation, phenotype clustering, CN identification and clinical analysis. **B** Representative IMC images (P61) displaying the staining of 17-marker antibodies in cancerous and paracancerous tissues. **C** Top: Combined tSNE plot illustrating that cells were grouped into 8 major cell types and 27 cell clusters in the cancerous and paracancerous tissues of ccRCC based on the expression of 17 markers, and are colored according to the cell cluster types. Bottom: tSNE plots showing the 27 cell clusters in cancerous and paracancerous tissues of ccRCC, respectively, and are colored according to patients. The letter A reflects the paracancerous tissue, the letter T reflects the cancerous tissue. **D** Heatmap illustrating the 17-marker expression profiles of the 27 cell clusters. Scale bar indicates the mean expression intensity. **E** Differences in cell components between cancerous and paracancerous tissues of ccRCC, including CD8^+^ T cells, Ki67^+^CD8^+^ T cells, Treg, CD163^−^ macrophages, endothelial cells, mesenchymal cells and fibroblasts

The workflow of the study is depicted in Fig. 1A. We used the Imaging Mass Cytometry (IMC) to explore the structural heterogeneity across ccRCC tissues and their association with clinical features and survival.

We combined 17-marker antibodies including the main immune cells and non-immune cells, to characterize the TME of ccRCC. For immune components, T cell- and macrophage-associated markers were selected because they are the main immune cells resident in ccRCC [24]. CD20 was also included due to its pivotal role in humoral immunity and TLS. Representative IMC images (patient 61 (P61)) showed the staining of marker antibodies. Markers such as αSMA, Pan-keratin, vimentin (Vim), CD31, etc. altered in density and spatial distribution between the cancerous and paracancerous tissues (Fig. 1B). To quantify the changes, we first extracted multi-plexed data of all cells from 13 cancerous and paired paracancerous tissues for dimensionality reduction, and finally identified 27 clusters (Fig. 1C, Additional file 1: Figure S1A, B). We further defined them as CD4^+^ T cells, CD8^+^ T cells, Ki67^+^CD8^+^ T cells, CD45RO^+^DP^+^ (double positive for CD4^+^CD8^+^) T cells, CD163^+^ macrophages, CD163^−^ macrophages, GZMB^+^ macrophages, B cells, regulatory T cells (Treg), epithelial cells, endothelial cells, mesenchymal cells, fibroblasts and other cells based on the expression profile of markers and tSNE in the 27 cell clusters (Fig. 1C, D, and Additional file 1: Figure S1C). Compared with the paracancerous tissues, the ccRCC tissues showed more CD8^+^ T, CD163^−^ macrophages, Treg cells, endothelial cells and fibroblasts, but less Ki67^+^CD8^+^ T cells and mesenchymal cells (Fig. 1E, Additional file 1: Figure S1D). It is worth noting that the difference in the level of cell components between cancerous and adjacent tissues also existed within the cancerous tissues.

## Heterogeneity of cell components in ccRCC tissues and its relationship with clinical survival

**Fig. 2:**
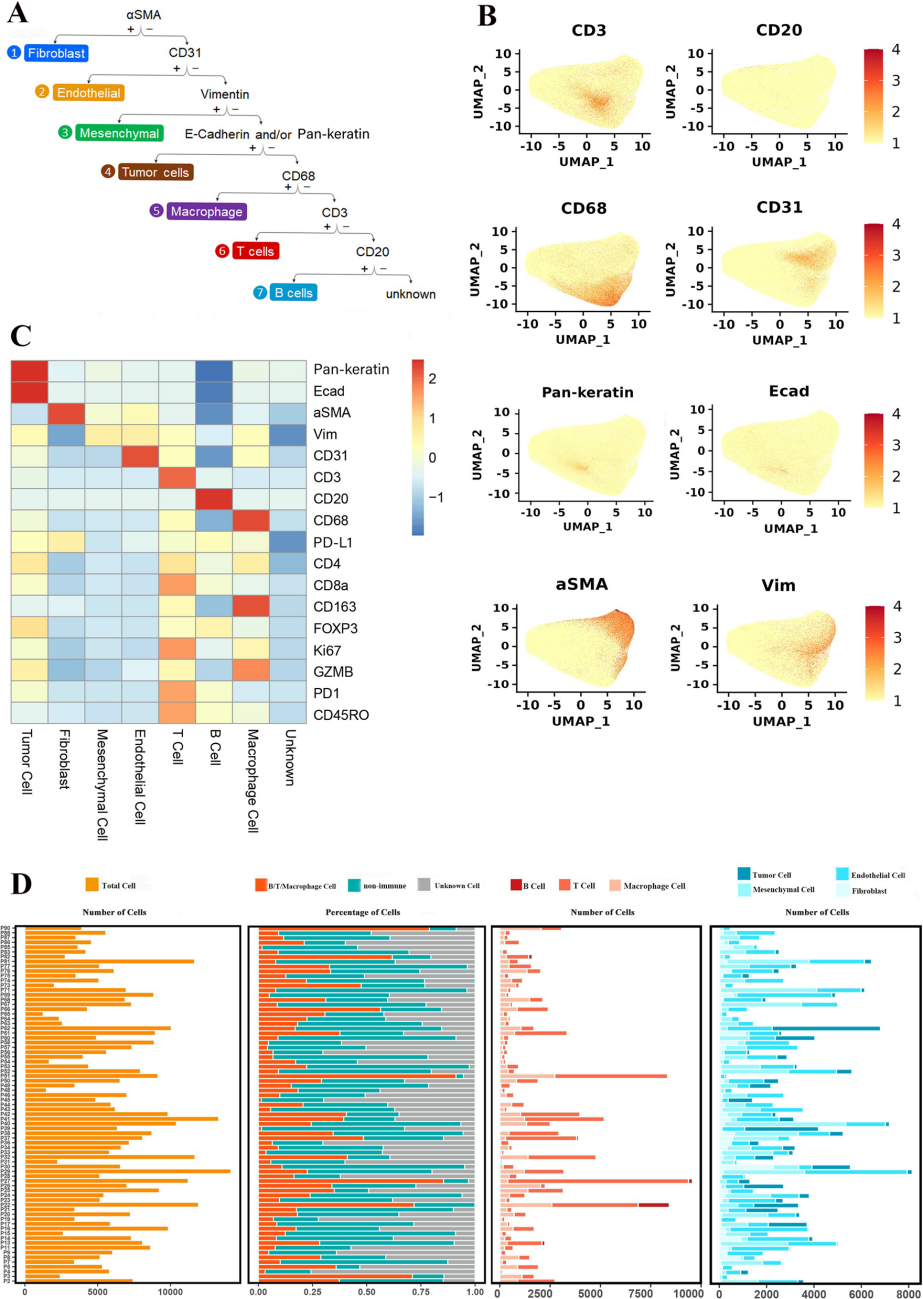
Heterogeneity of cell components in ccRCC tissues and its relationship with clinical survival. **A** Annotation rules of 7 major cell types, including T cells (CD3), macrophages (CD68), B cells (CD20), endothelial cells (CD31), fibroblasts (αSMA), mesenchymal cells (Vim) and tumor cells (Pan-keratin and/or Ecad), respectively. **B** UMAP maps colored by expression of CD3, CD68, CD20, CD31,αSMA, Vim, Pan-keratin and Ecad showing that cells from 75 ccRCC tissues. Scale bars indicate the normalized expression intensity. **C** Heatmap showing the 17-marker expression profiles across the 7 major cell types. Scale bar was normalized by z-score. **D** The proportion of different cell components and the number of 7 major cell types across various ccRCC tissues

To reveal the heterogeneous TME in ccRCC, we first used the UMAP to conduct dimensionality reduction for cells from 75 cancerous tissues. In total, we detected 468844 cells and used a supervised lineage assignment approach to identify 7 major cell types, including T cells (CD3), B cells (CD20), macrophages (CD68), endothelial cells (CD31), mesenchymal cells (Vim), fibroblasts (αSMA) and tumor cells (E-cadherin (Ecad), Pan-keratin) (Fig. 2A, B, Additional file 2: Figure S2B). The expression profile of 17 cell-specific markers in the 7 major cells is shown in Fig. 2C. Next, the number of the major cell types and the proportion of cell components were analyzed. The analysis of cell composition in each sample revealed that ccRCC is an immunologically and histologically heterogeneous tumor (Fig. 2D, Additional file 2: Figure S2A). However, none of the levels of immune or non-immune cells affects the survival of ccRCC patients (Additional file 2: Figure S2C).

## The immune cell distributions in the ccRCC tissues exhibited scattered and clustered status

**Fig. 3:**
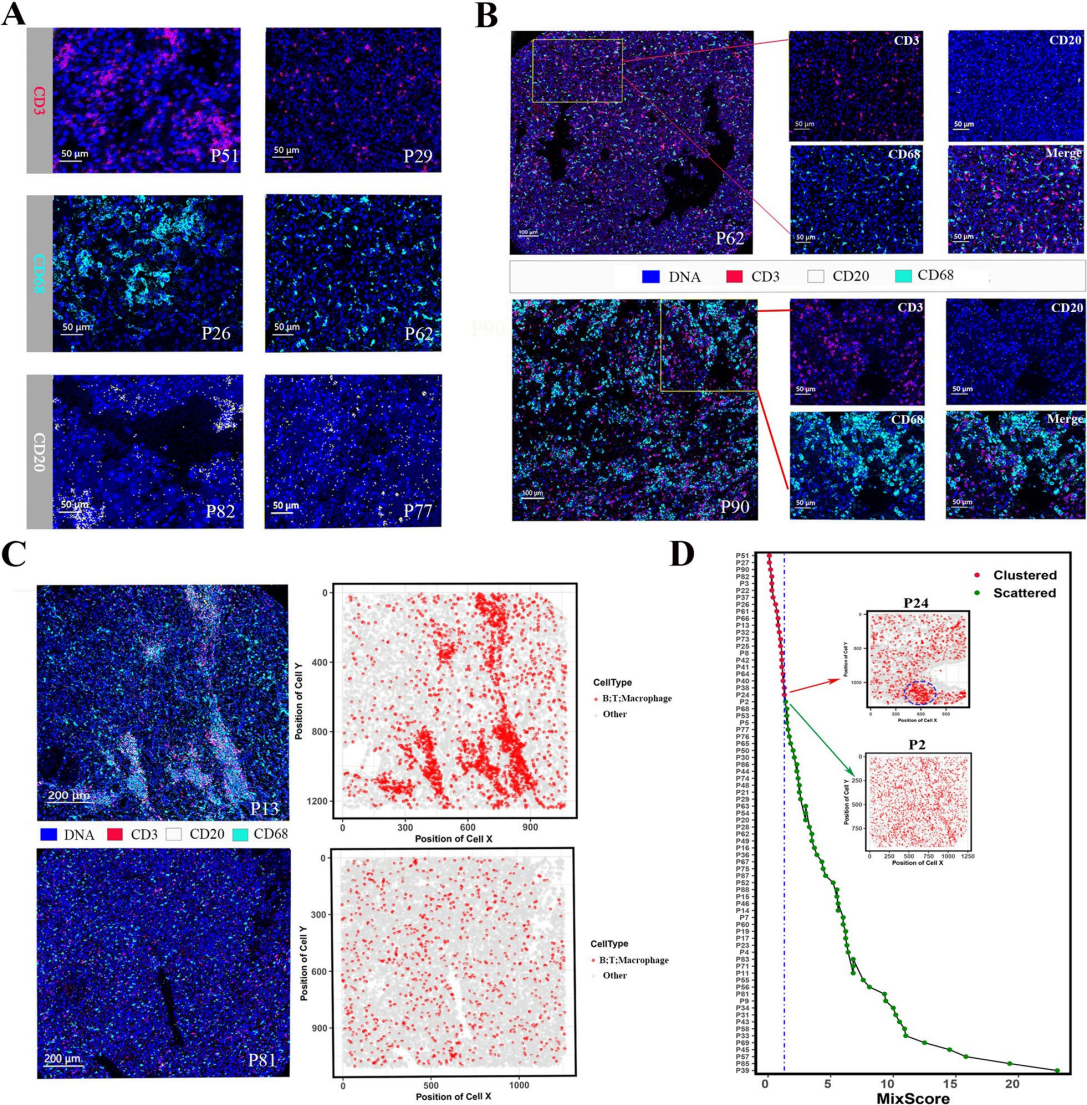
The immune cell distributions in the ccRCC tissues exhibited scattered and clustered status. **A** Representative IMC images depicting the clustered and scattered distributions of T cells (CD3), macrophages (CD68) and B cells (CD20). Clustered architecture in P51, P26 and P82 vs scattered architecture in P29, P62 and P77. **B** Representative IMC images illustrating the ccRCC tissues with scattered (Top panel: P62) and clustered (Bottom panel: P90) immune architectures. **C** IMC images and corresponding cell distribution maps of ccRCC tissues. Top panel: clustered architecture, P13; Bottom panel: scattered architecture, P81. **D** Mix score of each ccRCC tissues. The mix scores of P24 and P2 in the cohort were set as the thresholds of clustered and scattered immune architectures, respectively

Because different immune cells commonly exert functions through interactions, we were encouraged to investigate the heterogeneity in spatial structure other than the level of immune cell infiltrates. Whether T cells, macrophages or B cells, they all had clustered and scattered architectures in the ccRCC tissues (Fig. 3A). Regional microenvironment also exhibited two distinct immune architectures (Fig. 3B). To evaluate the spatial organization of tumor-immune in ccRCC, we first used the coordinate information of single cells extracted from each ccRCC tissue to generate cell distribution maps. The representative cell distribution maps (scattered, P81; clustered, P13) effectively capture the immune architecture observed in the corresponding IMC images (Fig. 3C). To assess the spatial proximity of T cells, B cells, macrophages, and other cells, we utilized the SPIAT [23] package of R to compute the mix score of each ccRCC tissue. In our cohort, the mix scores of P24 and P2 were set as the thresholds for distinguishing clustered and scattered groups due to their representative immune structures (Fig. 3D). The clustered group exhibited aggregated regions of immune cells (blue dotted circle), and the scattered group displayed a mix of tumor and immune cells. It is very attracive that the immune infiltrates in ccRCC tissue can be generally summarized as aggregation and dispersion patterns; however, they were not significantly related to the survival of patients (Additional file 3: Figure S3A).

We further evaluated five patients with metastatic ccRCC that were administrated sunitinib as the initial treatment after surgery (Additional file 6: Table S6). Three cases with scattered architecture responded to treatment, including two stable diseases (SD) and one partial response (PR). While the rest two cases with clustered architecture showed progressive disease (PD), one patient still experienced PD after being transferred to Axitinib + Pembrolizumab treatment, and the other patient died without receiving the follow-up treatment due to rapid PD (Additional file 5: Figure S5A).

## Cellular neighborhood (CN) analysis reflect the cell communities within the ccRCC tissues

**Fig. 4:**
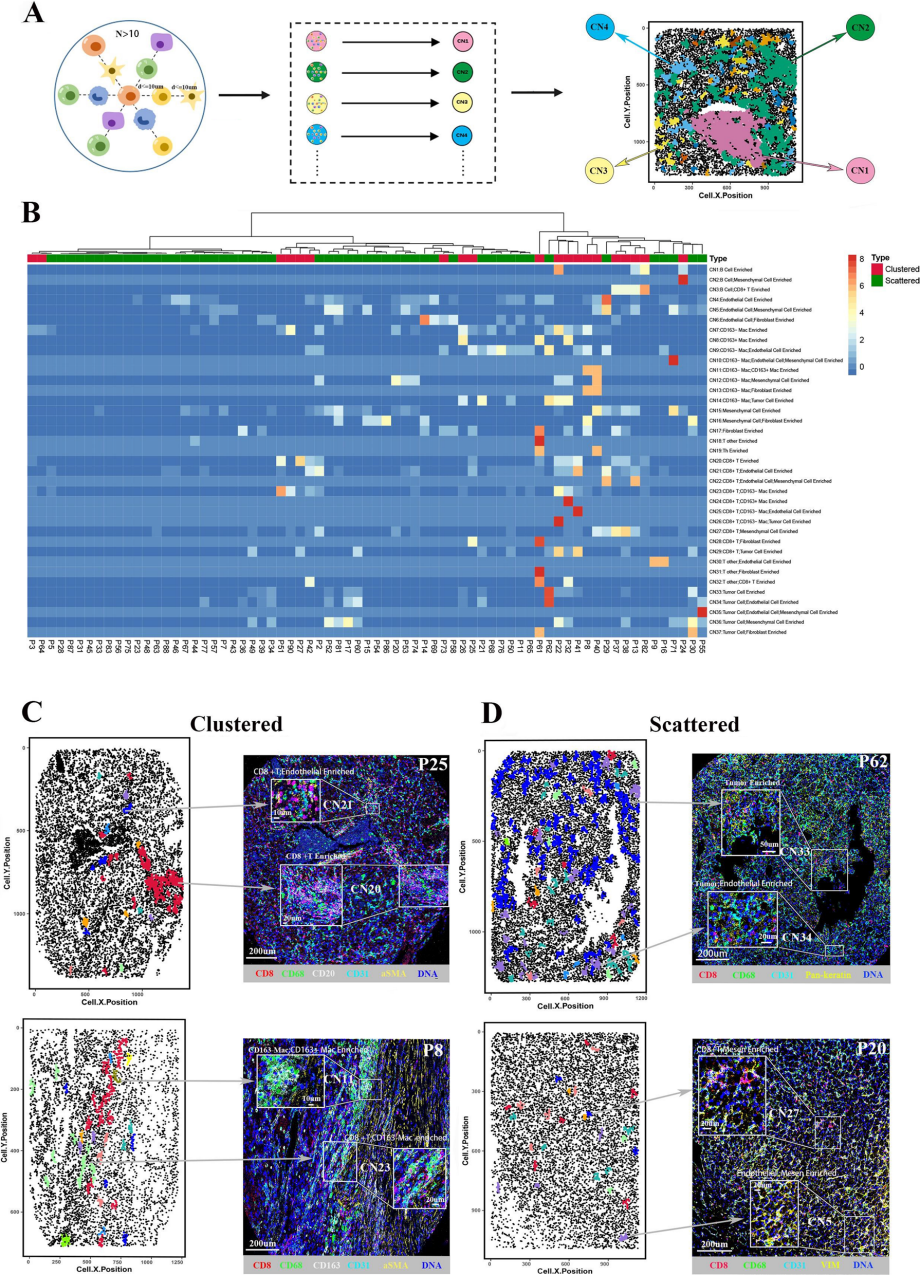
Cellular Neighborhood (CN) analysis reflect the cell communities within the ccRCC tissues. **A** Analysis schedule of CN. The topology of IMC image was represented by categorical dot plot with different colors indicating various CNs. **B** Heatmap showing the number of CNs across various ccRCC tissues. The numbers were normalized by z-score for visualization. **C** Categorical dot plots and corresponding IMC images of representative tissues with clustered immune architecture. Top: P25; Bottom: P8. **D** Categorical dot plots and corresponding IMC images of representative tissues with scattered immune architecture. Top: P62; Bottom: P20

To elucidate the functional units formed by cell-to-cell interactions, we used CN analysis to identify cell community in the regional microenvironment of ccRCC tissue. Schürch et al. [20] defined CN by the center cell and its 10 nearest neighbors. However, in order to remove artificial neighbors with separate cells in distance, we define CN (cell number > 10) as the center cell, its primary neighbors within 10 µm, plus the secondary neighbors of the primary neighbors within 10 µm (Fig. 4A). The numbers of various CNs in each ccRCC tissue were displayed in the heatmap (Fig. 4B, Additional file 2: Figure S2D). Interestingly, the CNs were also highly heterogeneous across different ccRCC tissues. The representative categorical dot plots and corresponding IMC images illustrated the ccRCC tissues with clustered (P2 and P25) and scattered (P14 and P71) immune architectures (Figs. 4C, D). The clustered group mainly has immune cell enriched CNs, such as CD8^+^ T cell CN, CD163^−^ macrophage_CD163^+^ macrophage CN and CD8^+^ T cell_CD163^−^ macrophage CN; while the scattered group is characterized by a large proportion of non-immune CNs, such as tumor cell CN, tumor cell_endothelial cell CN and endothelial cell_mesenchymal cell CN.

## TLS-like structure and macrophage/T cell communities in the clustered groups have distinct prognosis

**Fig. 5:**
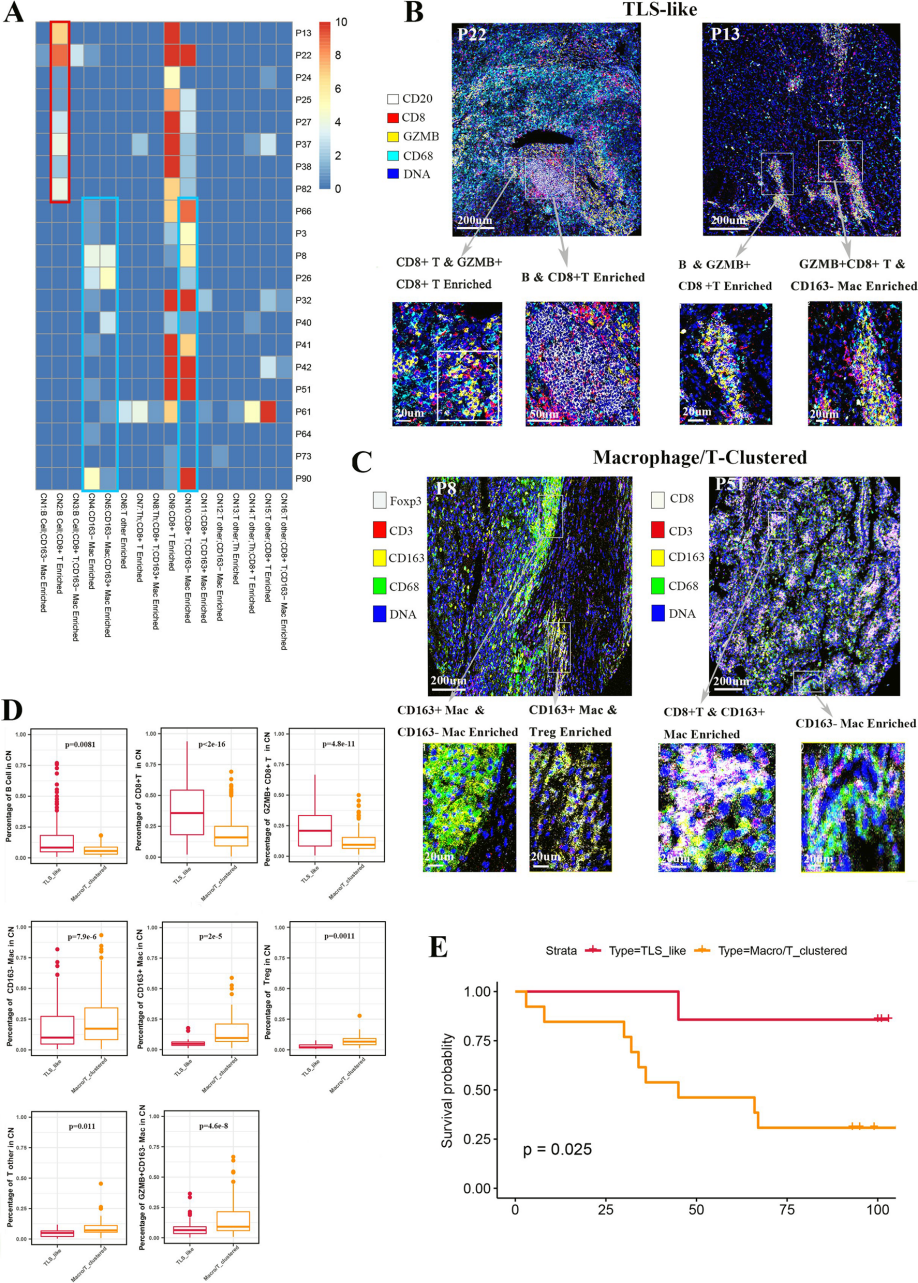
TLS-like structure and macrophage/T cell communities in the clustered groups has distinct prognosis. **A** Heatmap showing CN numbers across ccRCC tissues with clustered immune architecture. Red box denotes the TLS-like structure and blue box denotes the macrophage/T cell communities. Scale bar represents the number of CNs, with a maximum limit of 10. **B** Representative IMC images and characteristic CNs of the TLS-like phenotypes (P22 and P13). **C** Representative IMC images and characteristic CNs of the Macrophage/T-clustered phenotypes (P8 and P51). **D** Comparison of proportions of different cell types in CNs between the TLS-like and Macrophage/T-clustered phenotypes, including B cells, CD8^+^ T cells, GZMB^+^CD8^+^ T cells, CD163^−^ macrophages, CD163^+^ macrophages, Treg cells, T other cells and GZMB^+^CD163^−^ macrophages. **E** Comparison of survival between the TLS-like and Macrophage/T-clustered phenotypes

Based on the CN features of the clustered group, we identified two different phenotypes. A group of ccRCC tissues possessed cell communities consisting of T cells and B cells (red box), we defined them as the TLS-like phenotype due to its similarity to the reported TLS structure [13] (Fig. 5A). The TLS-like phenotype had more anti-tumor properties, which contained CD8^+^ T cell_GZMB^+^CD8^+^ T cell, B cell _CD8^+^ T cell, B cell_GZMB^+^CD8^+^ T cell and GZMB^+^CD8^+^ T cell_CD163^−^ macrophage CNs (Fig. 5B). The rest of the tissues, though immune clustered, contained no B cell community but were mainly characterized by T cell CN, macrophage CN or T cell_macrophage CN enrichment. We classified them as the macrophage/T-clustered phenotype. Compared with the TLS-like phenotype, the CNs of the macrophage/T-clustered phenotype were pro-tumor, such as macrophage, CD163^+^ macrophage_CD163^−^ macrophage and CD163^+^ macrophage_Treg CNs (Fig. 5A, C). We hypothesized that only the formation of CNs should better perform their physiological function by cell-to-cell interaction. Therefore, we analyzed the internal immune components in the CNs of the two phenotypes. The CNs of TLS-like phenotype had more B cells, CD8^+^ T cells and GZMB^+^CD8^+^ T cells, while the CNs of macrophage/T-clustered phenotype had more macrophages, T other cells and Treg cells (Fig. 5D, Additional file 2: Figure S2D). This explains why the TLS-like phenotype had better survival than the macrophage/T-clustered phenotype under similar clinical characteristics (Fig. 5E, Additional file 3: Figure S3B).

## Scattered group can be divided into immune-cold and -hot phenotypes

**Fig. 6:**
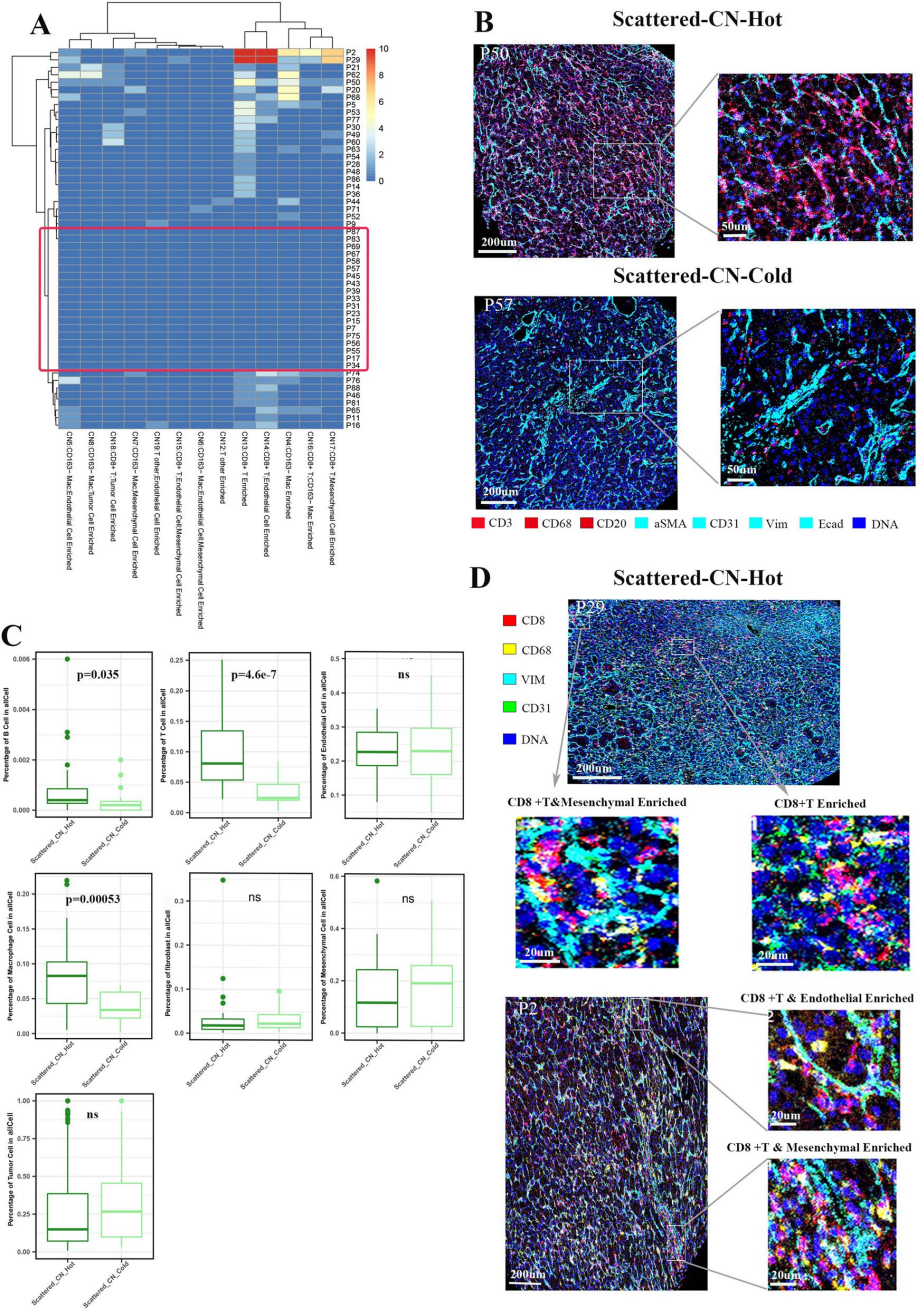
Scattered group can be divided into immune-cold and -hot phenotypes. **A** Unsupervised clustering of the ccRCC tissues with scattered immune architecture. Red box reflects the immune-cold phenotype. Scale bar represents the number of CNs, with a maximum limit of 10. **B** Spatial relationship between the immune (CD3, CD20 and CD68) and non-immune (αSMA, CD31, Vim and Ecad) components in the scattered-CN-cold and scattered-CN-hot phenotypes. **C** Comparison of the proportion of different cell types between the scattered-CN-cold and scattered-CN-hot phenotypes. **D** Representative IMC images of the scattered-CN-hot phenotype (P2 and P29). The zoomed areas display the characteristic CNs within the ccRCC tissues

In the scattered group, we found some tissues that had immune-associated CNs, while the rest (red box) didn’t, which were classified as scattered-CN-hot and scattered-CN-cold phenotypes, respectively (Fig. 6A, Additional file 4: Figure S4A). Compared with the scattered-CN-cold phenotype, the scattered-CN-hot phenotype was characterized by the spatial proximity of immune and non-immune components and higher proportions of B cells, T cells and macrophages (Fig. 6B, C). For the cell types in CNs, the scattered-CN-hot phenotype had more CD163^−^ macrophages and GZMB^+^CD8^+^ T cells, and fewer fibroblasts, mesenchymal cells and endothelial cells (Additional file 4: Figure S4B). The representative IMC images displayed a scattered-CN-hot ccRCC tissue with CD8^+^ T cell CN, CD8^+^ T cell_mesenchymal cell CN and CD8^+^ T cell_endothelial cell CN (Fig. 6D), and a scattered-CN-cold ccRCC tissue with mesenchymal cell CN, mesenchymal_endothelial cell CN, fibroblast CN and fibroblast_endothelial cell CN (Additional file 4: Figure S4C). The two phenotypes had no statistical significance in the survival and clinical characteristics (Additional file 3: Figure S3C). However, the survivals of the scattered-CN-cold and scattered-CN-hot phenotypes were superior to that of the macrophage/T-clustered group (Additional file 3: Figure S3D).

## The four phenotypes were associated with the clinical outcomes of ccRCC patients

**Fig. 7:**
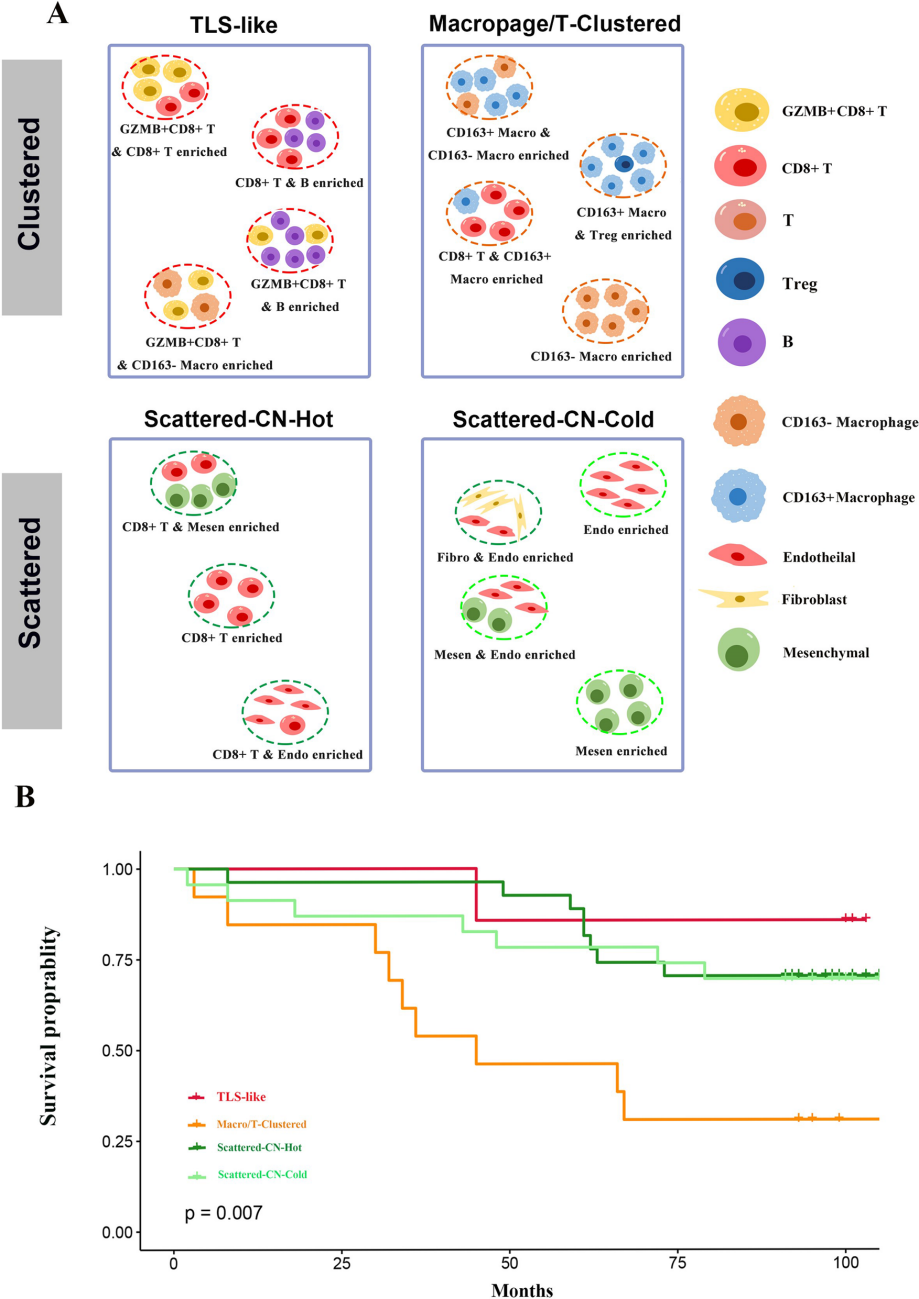
The four phenotypes were associated with the clinical outcomes of ccRCC patients. **A** The workflow of ccRCC patient stratification based on mix score and characteristic CNs. **B** Comparison of survival among the four phenotypes, including the TLS-like, macrophage/T-clustered, scattered-CN-cold and scattered-CN-hot phenotypes

Based on the spatial distributions and cell interactions of immune cells, we identified four phenotypes in ccRCC. The identification process is to first divide the tissues into clustered and scattered immune architectures according to their mix scores, and then conduct CN analysis considering both immune and non-immune components to determine the characteristic CNs in each tissue. The characteristic CNs of the four phenotypes are as follows: The TLS-like phenotype has GZMB^+^CD8^+^ T cell_B cell CN, CD8^+^ T_ B cell CN, GZMB^+^CD8^+^ T cell _CD8^+^ T cell CN or GZMB^+^CD8^+^ T_CD163^−^ macrophage CN; the Macrophage/T-clustered phenotype has CD163^+^ macrophage_CD163^−^ macrophage CN, CD163^+^ macrophage_Treg CN, CD8^+^ T_CD163^+^ macrophage CN or CD163^−^ macrophage CN; the scattered-CN-cold phenotype has non-immune CNs; the scattered-CN-hot phenotype has CD8^+^ T cell CN, CD8^+^ T cell_endothelial cell CN or CD8^+^ T cell_mesenchymal cell CN (Fig. 7A). The survival of the four phenotypes was significantly different (Fig. 7B), indicating that the functional units formed by cell communities have the potential to predict the prognosis of ccRCC patients.

The phenotype-specific CN rule was then applied to the five cases of metastatic ccRCC (Fig. 7A, Additional file 6: Table S6). Compared with the responders, the non-responders had more CD163^−^ macrophage_Tumor cell CN enrichment (Additional file 5: Figure S5B). The two non-responders (cases 4, 5) were classified as the macrophage/T-clustered phenotype, and their survival was inferior to that of three non-responsive cases (cases 1, 2, 3) belonging to the scattered-CN-hot phenotype (Additional file 5: Figure S5C, S5D, S5E). However, more samples are needed to validate the conclusion.

## Discussion

In this study, we discovered two distinct immune architectures in the ccRCC tissues. With the CN analysis, the ccRCC tissues were further divided into four prognostic TME phenotypes, which may additionally have potential in predicting the response to targeted therapy. These phenotypes may reflect the TME of ccRCC due to considering both immune and non-immune components, which will aid in more precise prognosis evaluation and personalized treatment.

With the rise of targeted therapy and immune-based therapy, traditional classifications of cancer patients based on ACJJ-TNM staging and pathological grading are showing limitations due to their inability to reflect the therapeutic response. Moreover, patients with the same clinical stage usually have various survival. For example, some patients with early stage ccRCC still have a metastatic risk after radical resection; the molecular mechanism underlying this is unknown, which easily leads to improper clinical decisions [25]. Exploring the TME features within ccRCC is helpful to identify early ccRCC patients with metastatic risk and optimize clinical therapeutics. In a retrospective study of 436 ccRCC cases, Ohe et al. [26] evaluated the immune score, three-tier and four-tier stratification systems. In contrast to other solid tumors, high-density CD8^+^ T cells is associated with poor survival in ccRCC [26–28]. Although these stratification systems are promising, they are all based on a simplified model of CD8^+^ T cell infiltration. The TME is a complex system in which B cells, Tregs and tumor associated macrophages are also potential immunotherapeutic targets of ccRCC [29–31]. Therefore, using only one immune cell type to stratify cancer patients is insufficient. An international tumor infiltrating lymphocytes (TILs) working group proposed a standardized visual assessment of H&E breast cancer sections that included more TILs [32]. Subsequently, the International lmmuno-Oncology Biomarkers Working Group applied the standardized TILs assessment to other solid tumors, including lung cancer, gastrointestinal cancer, urogenital cancer, etc. [33]. The above work improved the consistency and repeatability of TILs measurements. However, as they pointed out in the reviews, the standardized measurement has the following problems: 1. The evaluation area only covers stromal TILs but not the tumoral area; 2. It is difficult to evaluate TLS structure due to the diversity of TLS conformations and the complexity of immune cells. Considering that in recent years, some ccRCC stratification systems covering non-immune structures (such as vascular structure and cancer cell morphology) have also been proved to have high value in clinical management [34–36]. Therefore, to comprehensively evaluate the disease status of ccRCC patients, we should not only monitor the immune cells but also consider the non-immune cells and structural differences of TME in the tissues.

In this study, we used IMC, which can label multiple markers simultaneously at single-cell resolution while preserving spatial information, to obtain a high-dimensional image of ccRCC tissue [37–39]. In contrast to previously reported systems, we analyzed the whole ccRCC tissues without distinguishing TILs in intra- or peri-tumor [9, 32, 40]. We identified two immune architectures macroscopically in the ccRCC tissues. Although the same immune architectures were reported in liver cancer, their clinical significance has not been discussed [18]. Unfortunately, we didn’t observe any statistical significance in clinical characteristics or survival between the scattered and clustered groups. Therefore, we intended to further detail the intratumor cell communities associated with survival. Miheecheva et al. [41] reported that immune composition is conserved within each individual patient but profoundly different among patients using the multiregional analyses. This denotes that the CN analysis of a ccRCC tissue may be well patient-matched to identify the disease status. We finally discovered that based on the specific CNs, ccRCC tissues can be subdivided into four prognostic phenotypes, including the TLS-like, Macrophage/T-clustered, scattered-CN-cold and scattered-CN-hot phenotypes, respectively.

Although both Macrophage/T-clustered and TLS-like phenotypes belong to the TIME clustered structure, their distinct prognosis is determined by different CN units. The Macrophage/T-clustered phenotype represents the poorest survival prognosis among all TME phenotypes, which may be attributed to the presence of immunosuppressive cells such as CD163^+^ macrophages and Tregs. Additionally, the Macrophage/T-clustered phenotype is characterized by specific CN unit primarily composed of CD163^+^ macrophages. A similar phenomenon has been reported by Chakiryan et al. [42], linking macrophage aggregates to worsened survival prognosis in ccRCC patients. CD163 is used to identify tumor-associated macrophages (TAMs) in malignant diseases. In general, the levels of CD163^+^ TAMs correlate with poor OS and metastasis in malignant tumors, although the underlying mechanisms remain unclear. It is speculated that their strong anti-inflammatory effects may be one of the main reasons [43]. Additionally, the pro-angiogenic effects of CD163^+^ macrophages may also have an impact [44]. For example, in gastric cancer, CD163^+^ TAMs are significantly associated with increased microvessel density and worsened OS [45]. Furthermore, the Macrophage/T-clustered phenotype exhibits a kind of CN unit composed of CD163^+^ macrophages and CD8^+^ T cells. Braun et al. [46] observed the simultaneous enrichment of exhausted CD8^+^ T cells and inhibitory M2-like macrophages in advanced ccRCC tissues, along with restricted T cell receptor diversity. A study on liver cancer also found that the proximity of CD8^+^ T cells to arginase-1^high^ macrophages, rather than CD4^+^ T cells, is a salient feature of the TME in non-responders [19]. Therefore, we speculate that CD163^+^ macrophage-educated CD8^+^ T cells can lead to the immune suppressive or incompetent environment. The TLS-like phenotype is associated with a better survival prognosis, similar to previously reported TLS [13]. The reason for the favorable prognosis of the TLS-like phenotype may be attributed to the formation of specific cell communities, involving GZMB^+^CD8^+^ T cells, B cells, and CD163^−^ macrophages, which collectively induce anti-tumor effect. Although the TLS-like phenotype also consists of CN units composed of T cells and macrophages, the cell subtypes are GZMB^+^CD8^+^ T cells and CD163^−^ macrophages, rather than CD8^+^ T cells and CD163^+^ macrophages. CD8^+^ T cells expressing GZMB have been shown to play a central role in viral clearance and eradication of malignant cells through antigen-specific interactions with major histocompatibility complex class I-peptide complexes via T cell receptor [47]. Luo et al. [48] also found in an animal experiment on prostate cancer that increased GZMB^+^CD8^+^ T cells led to macrophage recruitment, primarily increasing tumor-killing M1 macrophages and reducing immunosuppressive M2 macrophages, thereby triggering anti-tumor immunity. Among the four phenotypes of ccRCC, we once considered whether scattered-CN-cold and scattered-CN-hot were different stages of the scattered group. But in Additional file 3: Figure S3C, we can observe the similar clinical stage composition between the scattered-CN-cold and the scattered-CN-hot phenotypes. Therefore, we ruled out the possibility that the scattered-CN-cold phenotype was formed due to less lymphocyte infiltration in the early stage of ccRCC. This indicates that evaluation based on TME structures can provide information beyond clinical staging. The survival of scattered-CN-cold patients is relatively favorable among the four TME phenotypes, similar to previous studies showing a correlation between low immune infiltration and improved survival in ccRCC [26, 49]. Notably, Xu et al. [49] demonstrated the poor response of immune-cold ccRCC to ICIs. This raises the question of whether targeting non-immune components could be the primary approach to enhance the prognosis of scattered-CN-cold patients [50, 51], given that non-immune CN is a characteristic feature of this phenotype. Although there was no difference in survival between the scattered-CN-cold and -hot phenotypes, they may differ in strategies to improve survival outcomes.

Subsequently, we observed that the metastatic lesions of patients with scattered-CN-hot phenotype were relatively well controlled after sunitinib administration, while the patients with macrophage/T-clustered phenotype had PD, even if one patient was converted to axitinib + pembrolizumab. This indicates the potential of immunophenotypes identified in this study to predict the response to targeted drugs and/or immunotherapy.

However, our study has some limitations. Firstly, we did not investigate other immune cells beyond T cells, B cells, and macrophages. As a result, when identifying cellular components in the CN units, we may have missed some aspects of the TME and cellular interactions. Secondly, the sample size in this study is still limited, particularly in terms of patients receiving immunotherapy, due to the following enrollment criteria: 1. Collected samples were required to have no prior exposure to targeted drugs or immunotherapy before nephrectomy to preserve the original TME of ccRCC; 2. Nephrectomy is typically not the first choice for patients with metastatic ccRCC. Future analysis should include larger and independent cohorts to ensure sufficient statistical power for identifying associations between clinical outcomes and the four TME phenotypes. Thirdly, the novelty of IMC technology imposes limitations on the analysis methods. Further development of analytical approaches is expected to enhance our understanding of spatial proteomics.

## Conclusion

In conclusion, we revealed four distinct immune phenotypes of ccRCC and identified their specific immune cell subtypes and CNs. These findings shed light on why some ccRCC patients with similar clinical features have different survival outcomes from the aspect of spatial heterogeneity of TME. This reminds us that even early ccRCC patients are necessary to receive more active clinical intervention if they belong to the Macrophage/T-clustered phenotype. More importantly, identifying the functional units formed by intercellular interaction may become a potential tool to evaluate prognosis and guide the ccRCC diagnosis and treatment.

## Supplementary Information

**Additional file 1: Figure S1.** (A) Combined tSNE plot illustrating the origin of the 27 cell clusters, which are colored according to tissues. The letter A reflects the paracancerous tissue, the letter T reflects the cancerous tissue. (B) Proportions of the 27 cell clusters in total cells from the 13 ccRCC and paired paracancerous tissues. (C) tSNE plots depicting the expression of 17 markers across the 27 cell clusters, respectively. (D) Comparison of the proportion of 27 cell clusters between the ccRCC and paired paracancerous tissues.

**Additional file 2: Figure S2.** (A) Heatmap representing the abundance of 7 major cells across different ccRCC tissues. The proportions were normalized by z-score for visualization. (B) Mean density curves showing the expression of 17 markers across total cells from 75 ccRCC tissues, respectively. The knee points are set as threshholds to determine the positive cells. (C) Comparison of survivals between high and low immune cell groups. High group, tissues with proportion of the immune cells ≥the third quartile (Q3); Low group, tissues with proportion of the immune cells ≤the first quartile (Q1). (D) Annotation rules for determining subtypes of T cells and macrophages.

**Additional file 3: Figure S3.** (A) Survival analysis between the scattered and clustered groups. (B) Comparison of clinical characteristics between the TLS-like and macrophage/T-clustered phenotypes. (C) Comparison of clinical characteristics between the scattered-CN-hot and scattered-CN-hot phenotypes. (D) Survival analysis between scattered-CN-cold, scattered-CN-hot and macrophage/T-clustered phenotypes.

**Additional file 4: Figure S4.** (A) Heatmap showing numbers of CNs across different ccRCC tissues. Scale bar represents the number of CNs, with a maximum limit of 10. (B) Comparison of the proportions of different cell components in CNs between the scattered-CN-cold and scattered-CN-hot phenotypes. (C) Representative IMC images showing the scattered-CN-cold ccRCC tissues (P15 and P14) with the characteristic CNs.

**Additional file 5: Figure S5.** (A) Heatmap depicting the various immune CNs across the 5 cases with metastatic ccRCC. (B) The difference in CNs between the responder and non-responder groups. (C) Representative IMC images of non-responder (cases 4 and 5) showing the macrophage/T-clustered characteristics. (D) Representative IMC images of responders (cases 1, 2 and3) displaying the scattered-CN-hot characteristics. (E) Survival analysis between the responder and non-responder groups.

**Additional file 6: Table S1.** Information of ccRCC tissue microarray. **Table S2.** Patient information for samples enrolled in the study (ccRCC tissue microarray). **Table S3.** Antibody panel used for acquiring images with IMC. **Table S4.** Details of IMC acquired images. **Table S5.** Cell type annotation rules. **Table S6. **Information of patients receiving targeted therapy and/or immunotherapy.

## Data Availability

The datasets used and/or analysed during the current study are available from the corresponding author on reasonable request.

## Abbreviations

ccRCC: Clear cell renal cell carcinoma
TME: Tumor microenvironment
TIME: Tumor immune microenvironment
ICI: Immune checkpoint inhibitor
IMC: Imaging mass cytometry
CN: Cellular neighborhood
DP: Double positive
GZMB: Granzyme B
Vim: Vimentin
Ecad: E-cadherin
αSMA: α-Smooth muscle actin
TLS: Tertiary lymphoid structure
TILs: Tumor infiltrating lymphocytes
PCA: Principal component analysis
UMAP: Uniform manifold approximation and projection

## References

[1] Siegel RL, Miller KD, Jemal A (2020). Cancer statistics. CA Cancer J Clin.

[2] Beck SD, Patel MI, Snyder ME (2004). Effect of papillary and chromophobe cell type on disease-free survival after nephrectomy for renal cell carcinoma. Ann Surg Oncol.

[3] Qu Y, Feng J, Wu X (2022). A proteogenomic analysis of clear cell renal cell carcinoma in a Chinese population. Nat Commun.

[4] D'Aniello C, Berretta M, Cavaliere C (2019). Biomarkers of prognosis and efficacy of anti-angiogenic therapy in metastatic clear cell renal cancer. Front Oncol.

[5] Motzer RJ, Escudier B, McDermott DF (2015). Nivolumab versus everolimus in advanced renal-cell carcinoma. N Engl J Med.

[6] Motzer RJ, Tannir NM, McDermott DF (2018). Nivolumab plus Ipilimumab versus sunitinib in advanced renal-cell carcinoma. N Engl J Med.

[7] Pal SK, McGregor B, Suarez C (2021). Cabozantinib in combination with atezolizumab for advanced renal cell carcinoma: results from the COSMIC-021 study. J Clin Oncol.

[8] Pages F, Mlecnik B, Marliot F (2018). International validation of the consensus Immunoscore for the classification of colon cancer: a prognostic and accuracy study. Lancet.

[9] Galon J, Bruni D (2019). Approaches to treat immune hot, altered and cold tumours with combination immunotherapies. Nat Rev Drug Discov.

[10] Obradovic A, Chowdhury N, Haake SM (2021). Single-cell protein activity analysis identifies recurrence-associated renal tumor macrophages. Cell.

[11] Wang J, Huang F, Zhao J (2022). Tumor-infiltrated CD8+ T Cell 10-gene signature related to clear cell renal cell carcinoma prognosis. Front Immunol.

[12] Yang F, Zhao J, Luo X (2021). Transcriptome profiling reveals B-lineage cells contribute to the poor prognosis and metastasis of clear cell renal cell carcinoma. Front Oncol.

[13] Meylan M, Petitprez F, Becht E (2022). Tertiary lymphoid structures generate and propagate anti-tumor antibody-producing plasma cells in renal cell cancer. Immunity.

[14] Helmink BA, Reddy SM, Gao J (2020). B cells and tertiary lymphoid structures promote immunotherapy response. Nature.

[15] Ruffin AT, Cillo AR, Tabib T (2021). B cell signatures and tertiary lymphoid structures contribute to outcome in head and neck squamous cell carcinoma. Nat Commun.

[16] Kroeger DR, Milne K, Nelson BH (2016). Tumor-infiltrating plasma cells are associated with tertiary lymphoid structures, cytolytic T-cell responses, and superior prognosis in ovarian cancer. Clin Cancer Res.

[17] Keren L, Bosse M, Marquez D (2018). A structured tumor-immune microenvironment in triple negative breast cancer revealed by multiplexed ion beam imaging. Cell.

[18] Sheng J, Zhang J, Wang L (2022). Topological analysis of hepatocellular carcinoma tumour microenvironment based on imaging mass cytometry reveals cellular neighbourhood regulated reversely by macrophages with different ontogeny. Gut.

[19] Mi H, Ho WJ, Yarchoan M (2022). Multi-scale spatial analysis of the tumor microenvironment reveals features of cabozantinib and nivolumab efficacy in hepatocellular carcinoma. Front Immunol.

[20] Schurch CM, Bhate SS, Barlow GL (2020). Coordinated cellular neighborhoods orchestrate antitumoral immunity at the colorectal cancer invasive front. Cell.

[21] Goltsev Y, Samusik N, Kennedy-Darling J (2018). Deep profiling of mouse splenic architecture with CODEX multiplexed imaging. Cell.

[22] Schapiro D, Jackson HW, Raghuraman S (2017). histoCAT: analysis of cell phenotypes and interactions in multiplex image cytometry data. Nat Methods..

[23] Feng Y, Yang T, Zhu J (2023). Spatial analysis with SPIAT and spaSim to characterize and simulate tissue microenvironments. Nat Commun.

[24] Chevrier S, Levine JH, Zanotelli VRT (2017). An Immune atlas of clear cell renal cell carcinoma. Cell.

[25] Ma X, Gu L, Li H (2015). Hypoxia-induced overexpression of stanniocalcin-1 is associated with the metastasis of early stage clear cell renal cell carcinoma. J Transl Med.

[26] Ohe C, Yoshida T, Ikeda J (2022). Histologic-based tumor-associated immune cells status in clear cell renal cell carcinoma correlates with gene signatures related to cancer immunity and clinical outcomes. Biomedicines.

[27] Nakano O, Sato M, Naito Y (2001). Proliferative activity of intratumoral CD8(+) T-lymphocytes as a prognostic factor in human renal cell carcinoma: clinicopathologic demonstration of antitumor immunity. Cancer Res.

[28] Giraldo NA, Becht E, Vano Y (2017). Tumor-infiltrating and peripheral blood T-cell immunophenotypes predict early relapse in localized clear cell renal cell carcinoma. Clin Cancer Res.

[29] Kim MC, Borcherding N, Ahmed KK (2021). CD177 modulates the function and homeostasis of tumor-infiltrating regulatory T cells. Nat Commun.

[30] Cowman SJ, Fuja DG, Liu XD (2020). Macrophage HIF-1alpha Is an independent prognostic indicator in kidney cancer. Clin Cancer Res.

[31] Carril-Ajuria L, Desnoyer A, Meylan M (2022). Baseline circulating unswitched memory B cells and B-cell related soluble factors are associated with overall survival in patients with clear cell renal cell carcinoma treated with nivolumab within the NIVOREN GETUG-AFU 26 study. J Immunother Cancer.

[32] Salgado R, Denkert C, Demaria S (2015). The evaluation of tumor-infiltrating lymphocytes (TILs) in breast cancer: recommendations by an International TILs Working Group 2014. Ann Oncol.

[33] Hendry S, Salgado R, Gevaert T (2017). Assessing tumor-infiltrating lymphocytes in solid tumors: a practical review for pathologists and proposal for a standardized method from the international immuno-oncology biomarkers working group: part 2: TILs in melanoma, gastrointestinal tract carcinomas, non-small cell lung carcinoma and mesothelioma, endometrial and ovarian carcinomas, squamous cell carcinoma of the head and neck, genitourinary carcinomas, and primary brain tumors. Adv Anat Pathol.

[34] Ohe C, Yoshida T, Amin MB (2022). Development and validation of a vascularity-based architectural classification for clear cell renal cell carcinoma: correlation with conventional pathological prognostic factors, gene expression patterns, and clinical outcomes. Mod Pathol.

[35] Verine J, Colin D, Nheb M (2018). Architectural patterns are a relevant morphologic grading system for clear cell renal cell carcinoma prognosis assessment: comparisons with WHO/ISUP grade and integrated staging systems. Am J Surg Pathol.

[36] Cai Q, Christie A, Rajaram S (2020). Ontological analyses reveal clinically-significant clear cell renal cell carcinoma subtypes with convergent evolutionary trajectories into an aggressive type. EBioMedicine.

[37] Guo N, van Unen V, Ijsselsteijn ME (2020). A 34-marker panel for imaging mass cytometric analysis of human snap-frozen tissue. Front Immunol.

[38] Zhang Y, Wang Y, Cao WW (2018). Spectral characteristics of autofluorescence in renal tissue and methods for reducing fluorescence background in confocal laser scanning microscopy. J Fluoresc.

[39] Bertocchi A, Carloni S, Ravenda PS (2021). Gut vascular barrier impairment leads to intestinal bacteria dissemination and colorectal cancer metastasis to liver. Cancer Cell.

[40] Hegde PS, Chen DS (2020). Top 10 challenges in cancer immunotherapy. Immunity.

[41] Miheecheva N, Postovalova E, Lyu Y (2022). Multiregional single-cell proteogenomic analysis of ccRCC reveals cytokine drivers of intratumor spatial heterogeneity. Cell Rep.

[42] Chakiryan NH, Kimmel GJ, Kim Y (2021). Spatial clustering of CD68+ tumor associated macrophages with tumor cells is associated with worse overall survival in metastatic clear cell renal cell carcinoma. PLoS ONE.

[43] Skytthe MK, Graversen JH, Moestrup SK (2020). Targeting of CD163(+) macrophages in inflammatory and malignant diseases. Int J Mol Sci.

[44] Guo L, Akahori H, Harari E (2018). CD163+ macrophages promote angiogenesis and vascular permeability accompanied by inflammation in atherosclerosis. J Clin Invest.

[45] Park JY, Sung JY, Lee J (2016). Polarized CD163+ tumor-associated macrophages are associated with increased angiogenesis and CXCL12 expression in gastric cancer. Clin Res Hepatol Gastroenterol.

[46] Braun DA, Street K, Burke KP (2021). Progressive immune dysfunction with advancing disease stage in renal cell carcinoma. Cancer Cell.

[47] Jonsson AH, Zhang F, Dunlap G (2022). Granzyme K(+) CD8 T cells form a core population in inflamed human tissue. Sci Transl Med.

[48] Luo ZW, Xia K, Liu YW (2021). Extracellular vesicles from akkermansia muciniphila elicit antitumor immunity against prostate cancer via modulation of CD8(+) T cells and macrophages. Int J Nanomedicine.

[49] Xu W, Anwaier A, Ma C (2021). Prognostic immunophenotyping clusters of clear cell renal cell carcinoma defined by the unique tumor immune microenvironment. Front Cell Dev Biol.

[50] Ambrosetti D, Coutts M, Paoli C (2022). Cancer-associated fibroblasts in renal cell carcinoma: implication in prognosis and resistance to anti-angiogenic therapy. BJU Int.

[51] Wuttig D, Zastrow S, Fussel S (2012). CD31, EDNRB and TSPAN7 are promising prognostic markers in clear-cell renal cell carcinoma revealed by genome-wide expression analyses of primary tumors and metastases. Int J Cancer.

