# Supplementary figures and images for: Spatial heterogeneity of tumor microenvironment influences the prognosis of clear cell renal cell carcinoma
Source: J Transl Med. 2023 Jul 20;21:489. doi: 10.1186/s12967-023-04336-8 (PMC10360235; doi:10.1186/s12967-023-04336-8)

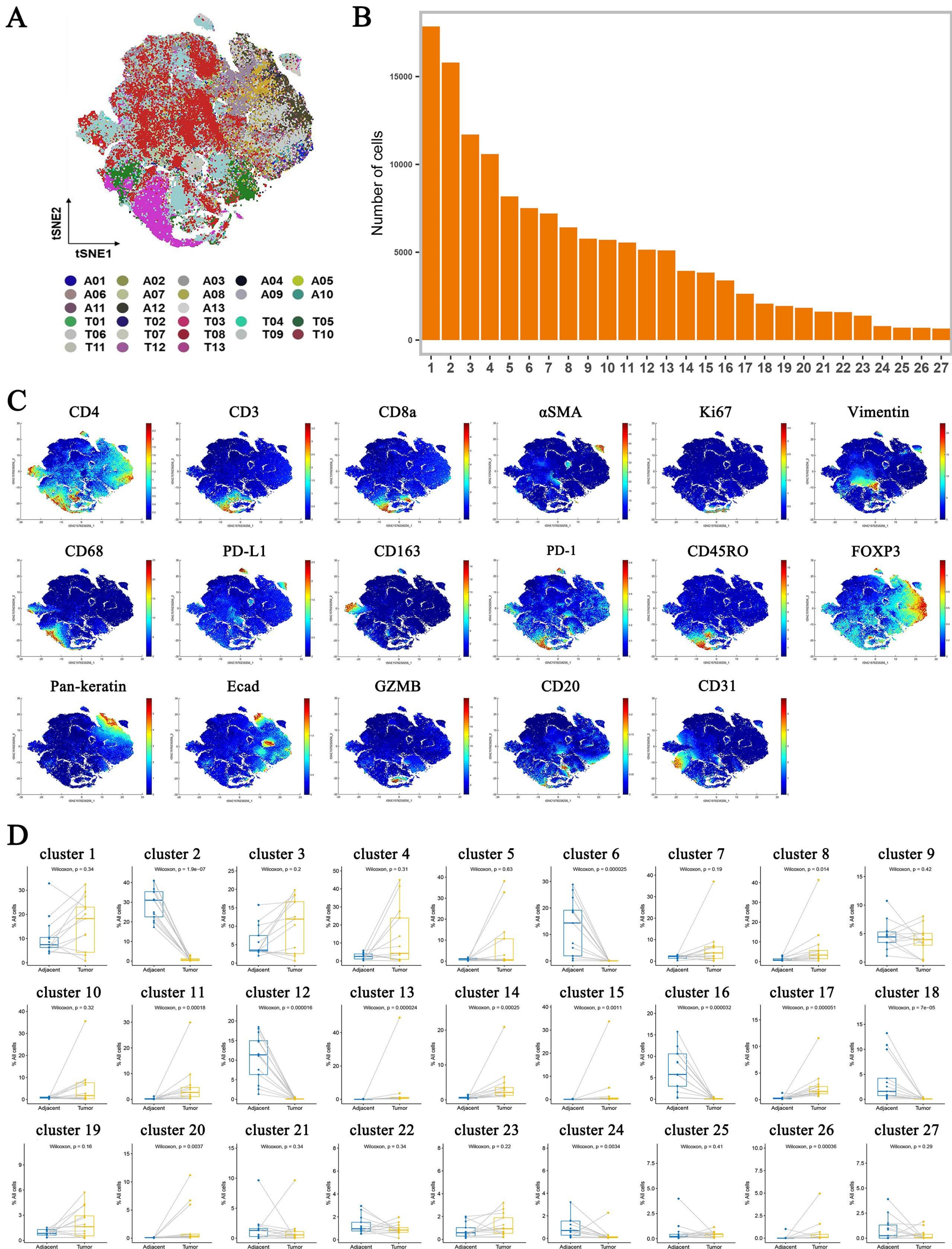

Figure S1

Supplement: Supplementary file 1 — Additional file 1: Figure S1. (A) Combined tSNE plot illustrating the origin of the 27 cell clusters, which are colored according to tissues. The letter A reflects the paracancerous tissue, the letter T reflects the cancerous tissue. (B) Proportions of the 27 cell clusters in total cells from the 13 ccRCC and paired paracancerous tissues. (C) tSNE plots depicting the expression of 17 markers across the 27 cell clusters, respectively. (D) Comparison of the proportion of 27 cell clusters between the ccRCC and paired paracancerous tissues. [file 12967_2023_4336_MOESM1_ESM.pdf]

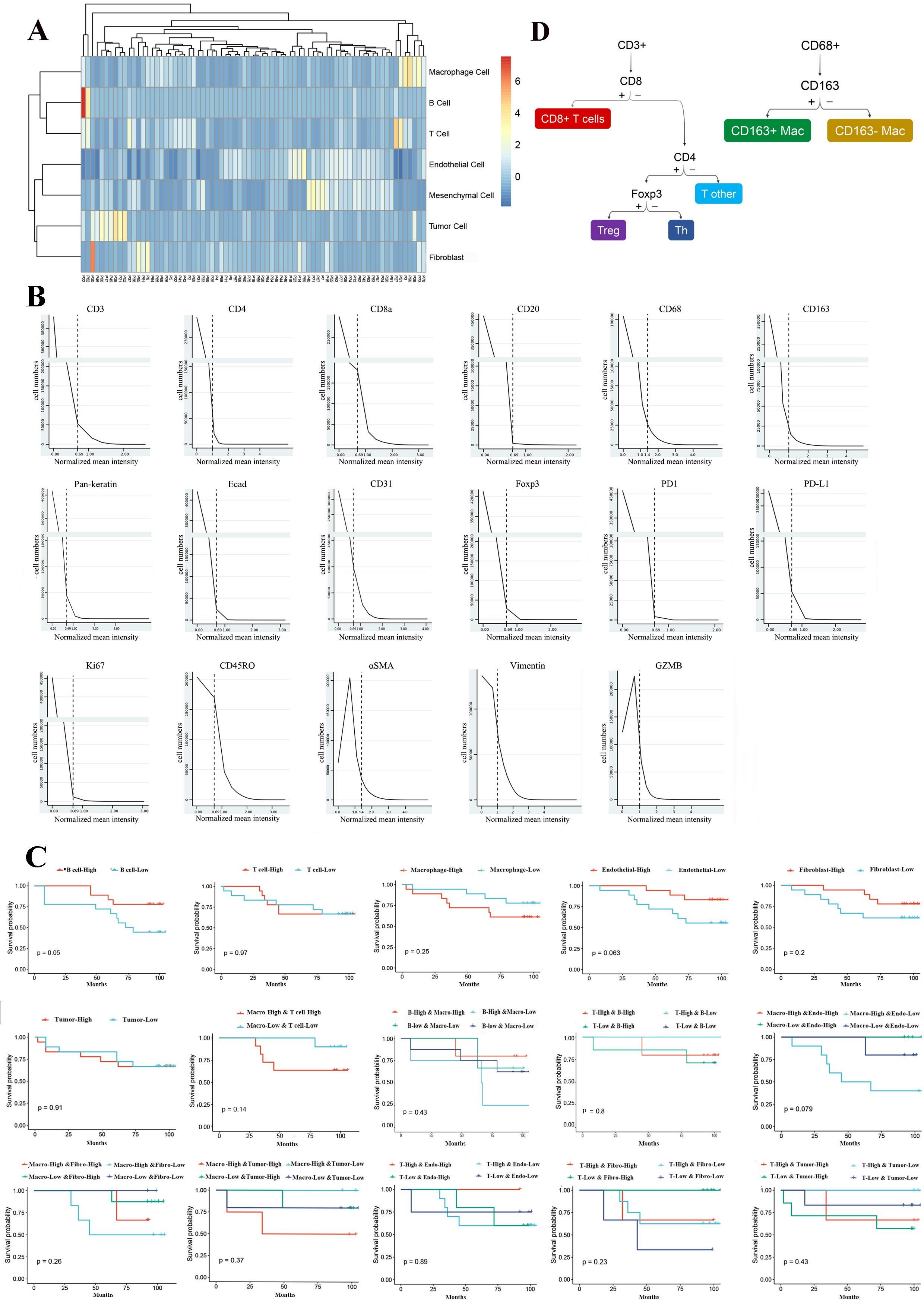

Supplement: Supplementary file 2 — Additional file 2: Figure S2. (A) Heatmap representing the abundance of 7 major cells across different ccRCC tissues. The proportions were normalized by z-score for visualization. (B) Mean density curves showing the expression of 17 markers across total cells from 75 ccRCC tissues, respectively. The knee points are set as threshholds to determine the positive cells. (C) Comparison of survivals between high and low immune cell groups. High group, tissues with proportion of the immune cells ≥the third quartile (Q3); Low group, tissues with proportion of the immune cells ≤the first quartile (Q1). (D) Annotation rules for determining subtypes of T cells and macrophages. [file 12967_2023_4336_MOESM2_ESM.pdf]

A

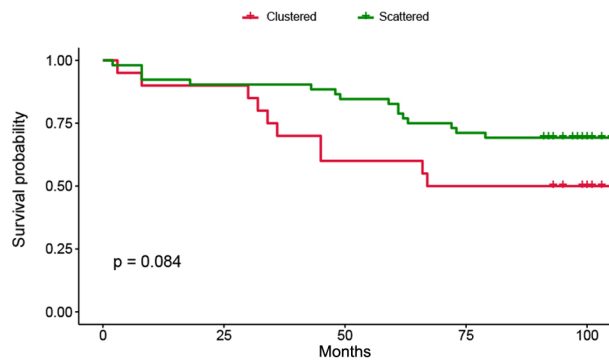

B

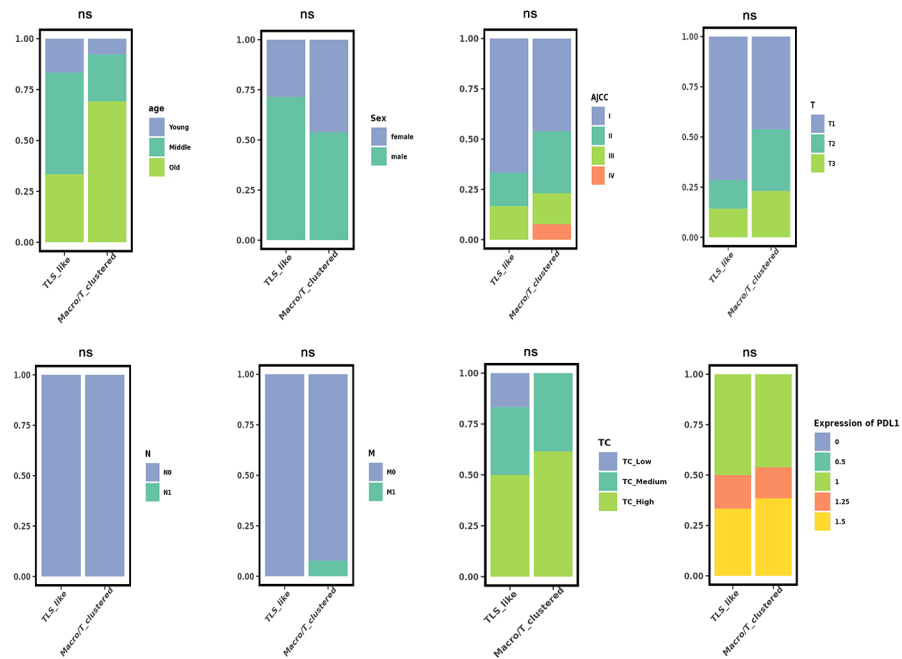

C

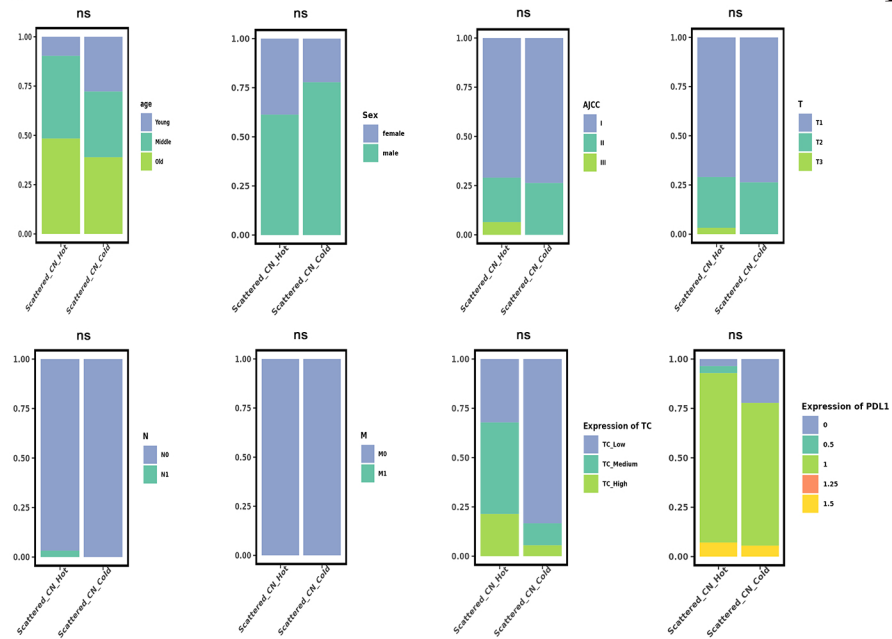

D

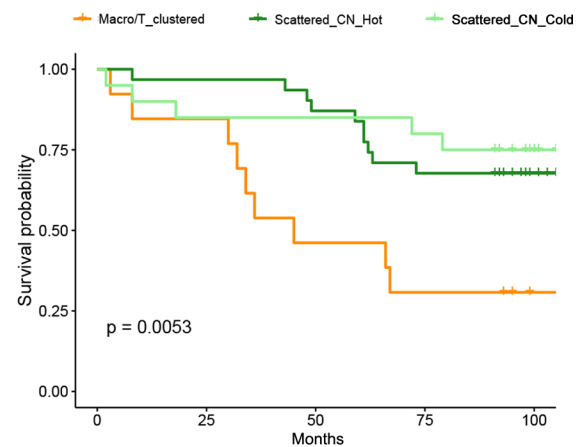

Figure S3

Supplement: Supplementary file 3 — Additional file 3: Figure S3. (A) Survival analysis between the scattered and clustered groups. (B) Comparison of clinical characteristics between the TLS-like and macrophage/T-clustered phenotypes. (C) Comparison of clinical characteristics between the scattered-CN-hot and scattered-CN-hot phenotypes. (D) Survival analysis between scattered-CN-cold, scattered-CN-hot and macrophage/T-clustered phenotypes. [file 12967_2023_4336_MOESM3_ESM.pdf]

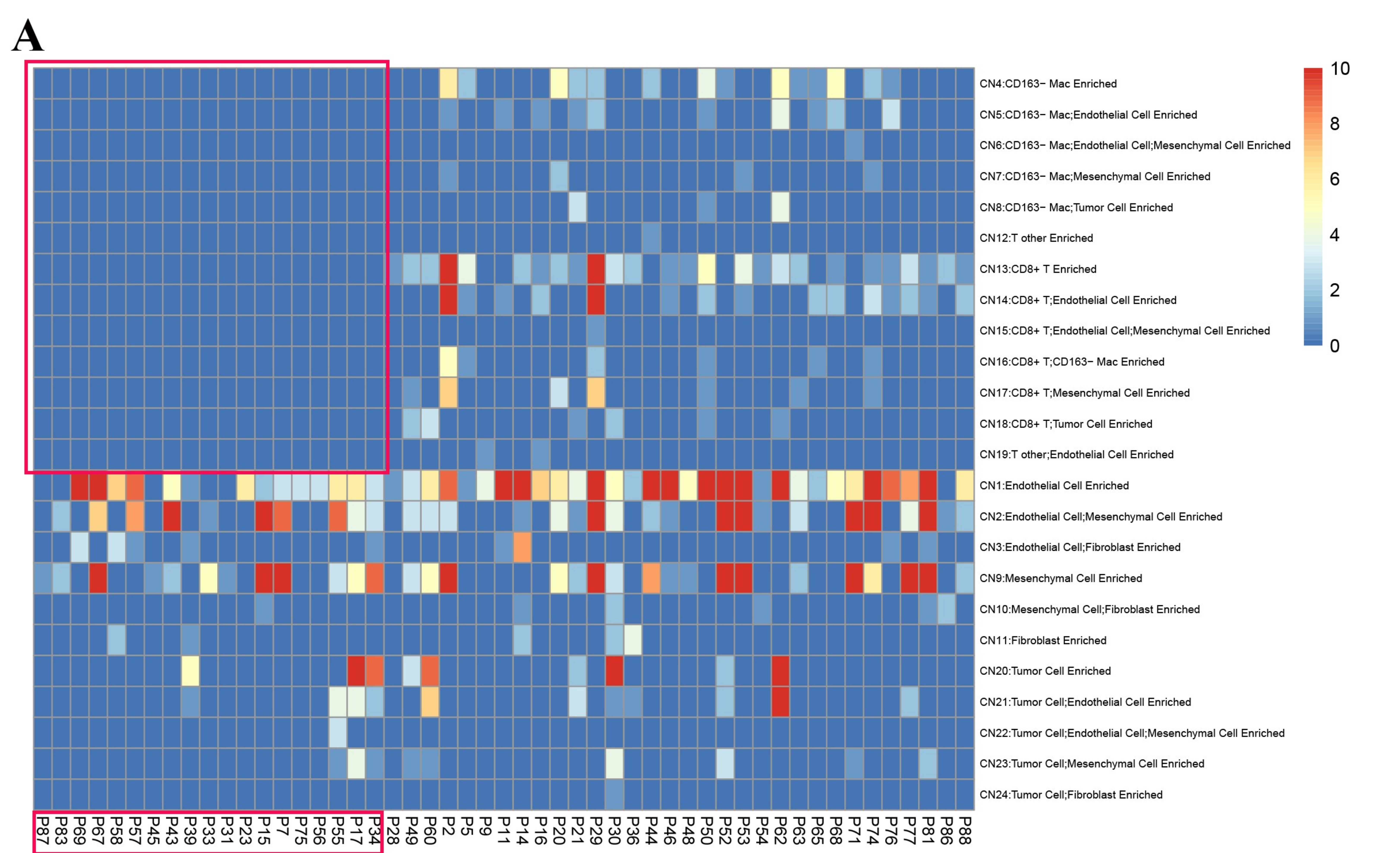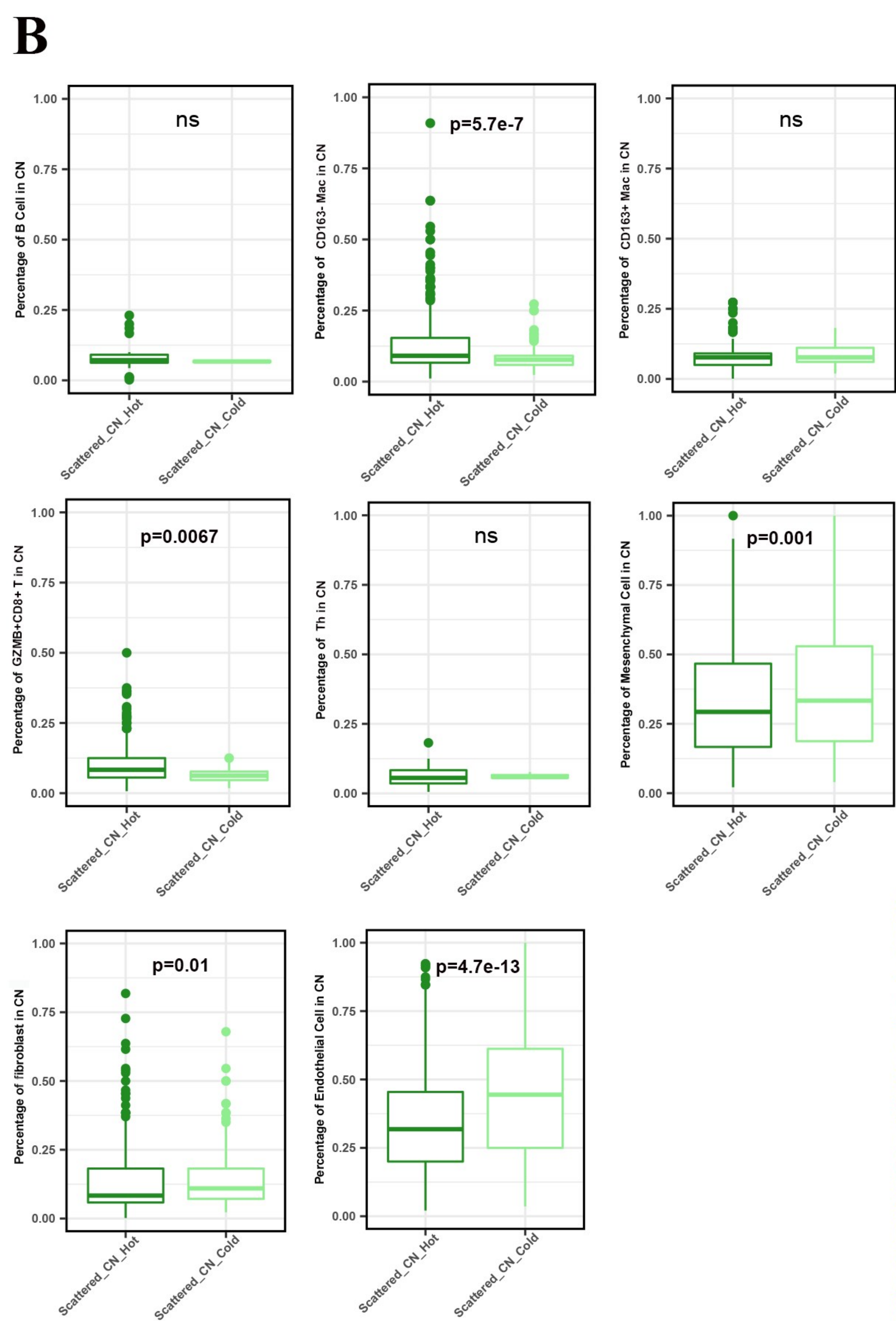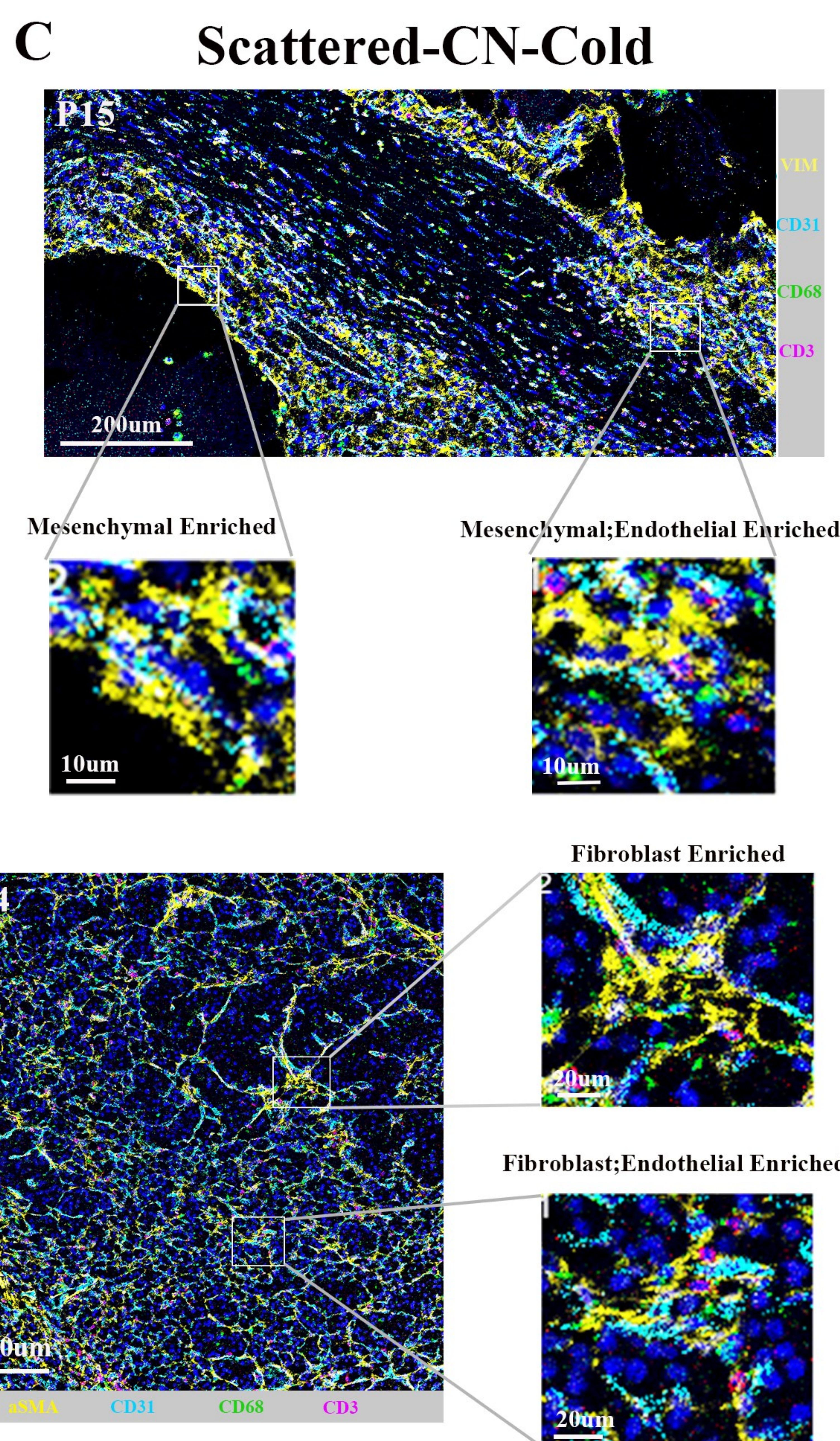

Figure S4

Supplement: Supplementary file 4 — Additional file 4: Figure S4. (A) Heatmap showing numbers of CNs across different ccRCC tissues. Scale bar represents the number of CNs, with a maximum limit of 10. (B) Comparison of the proportions of different cell components in CNs between the scattered-CN-cold and scattered-CN-hot phenotypes. (C) Representative IMC images showing the scattered-CN-cold ccRCC tissues (P15 and P14) with the characteristic CNs. [file 12967_2023_4336_MOESM4_ESM.pdf]

A

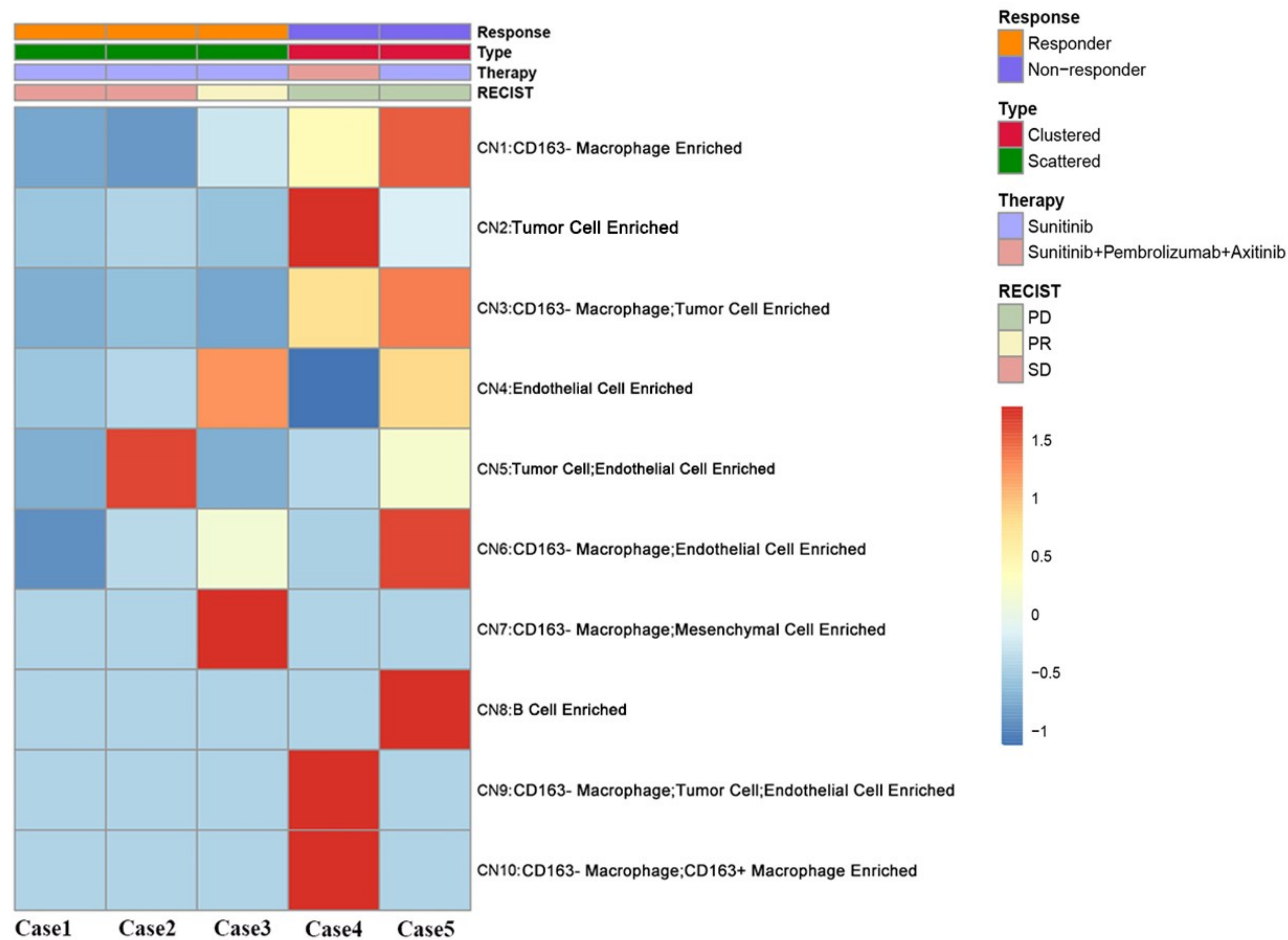

B

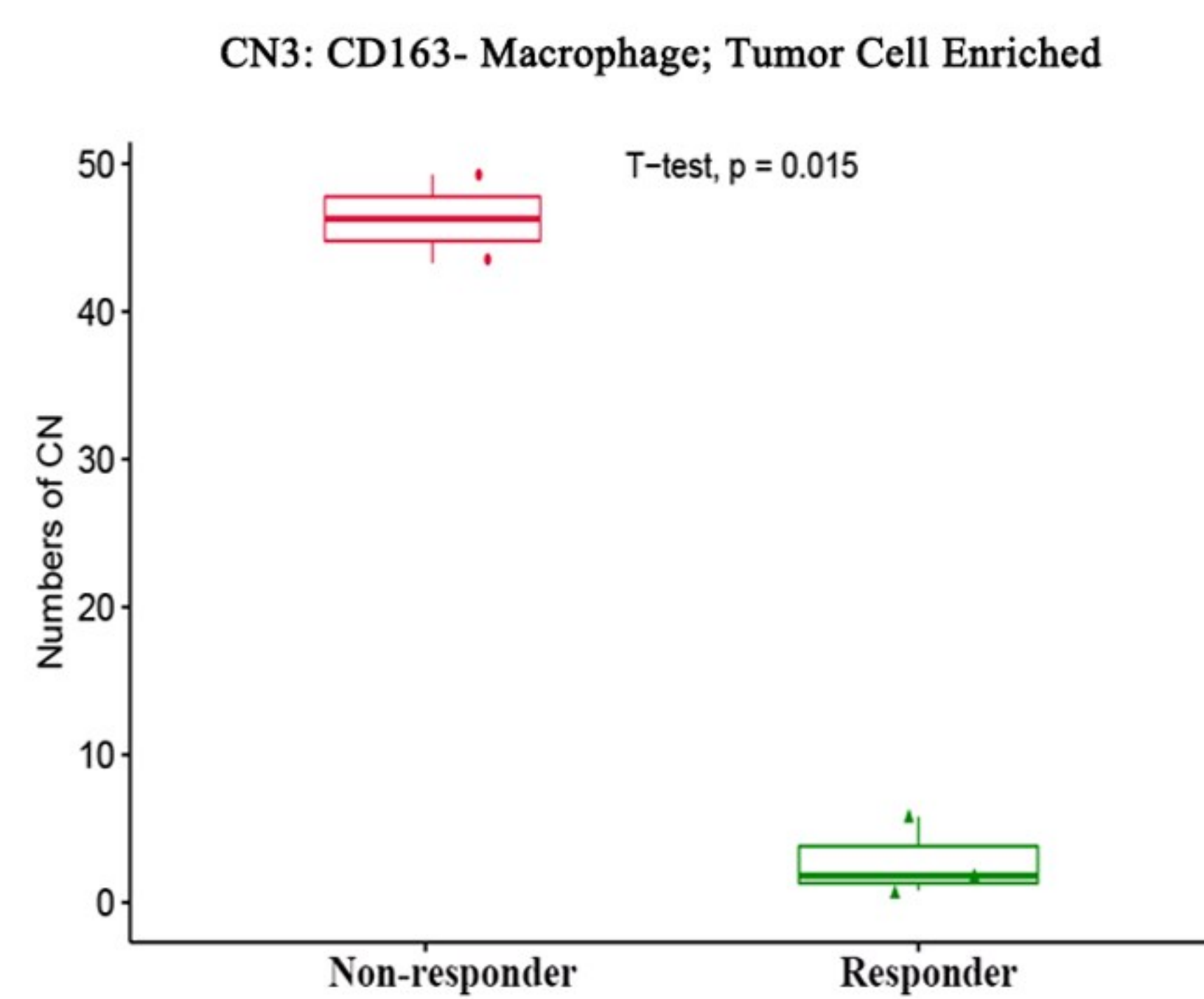

D

## Scattered-CN-Hot

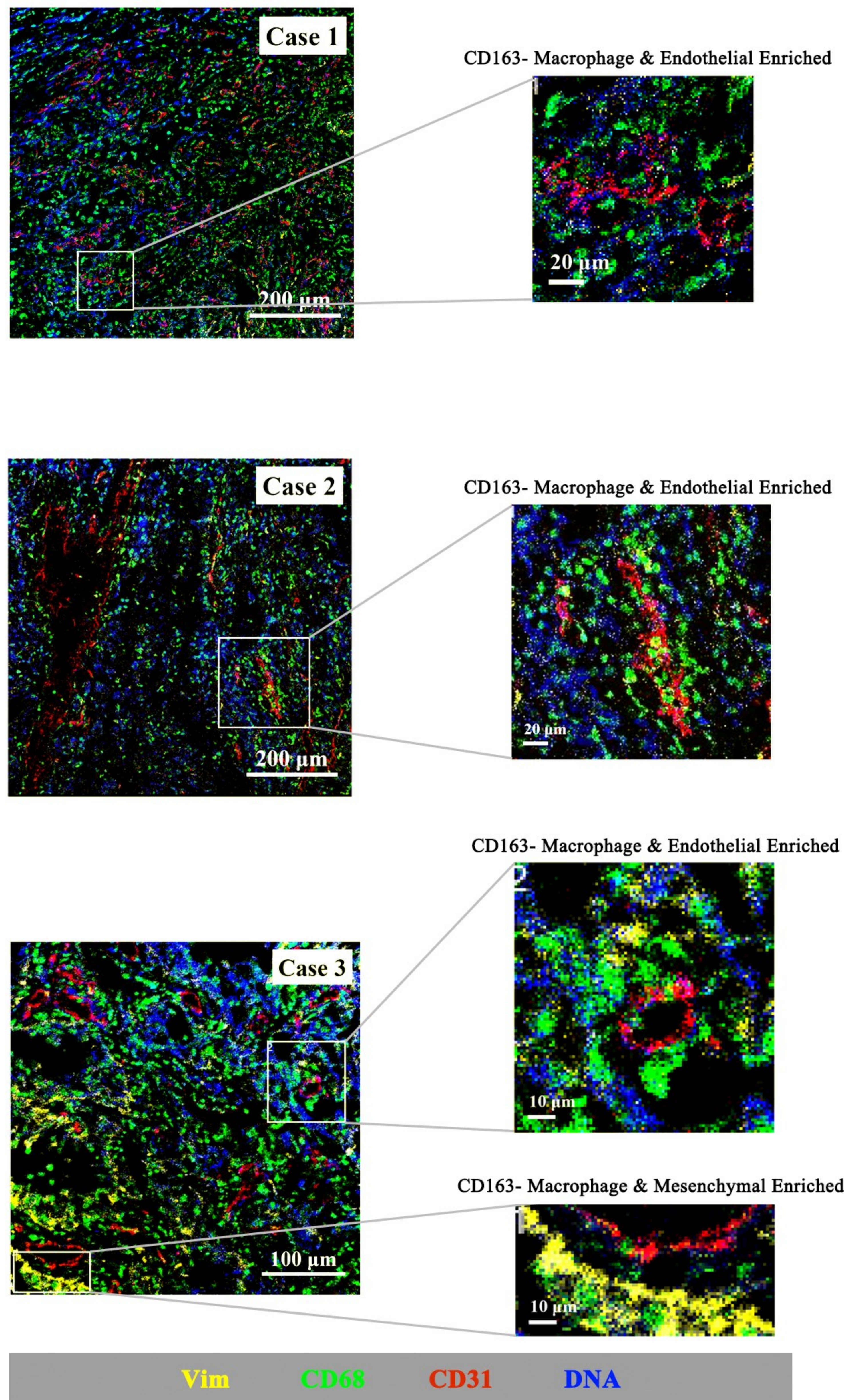

C

## Macrophage/T-Clustered

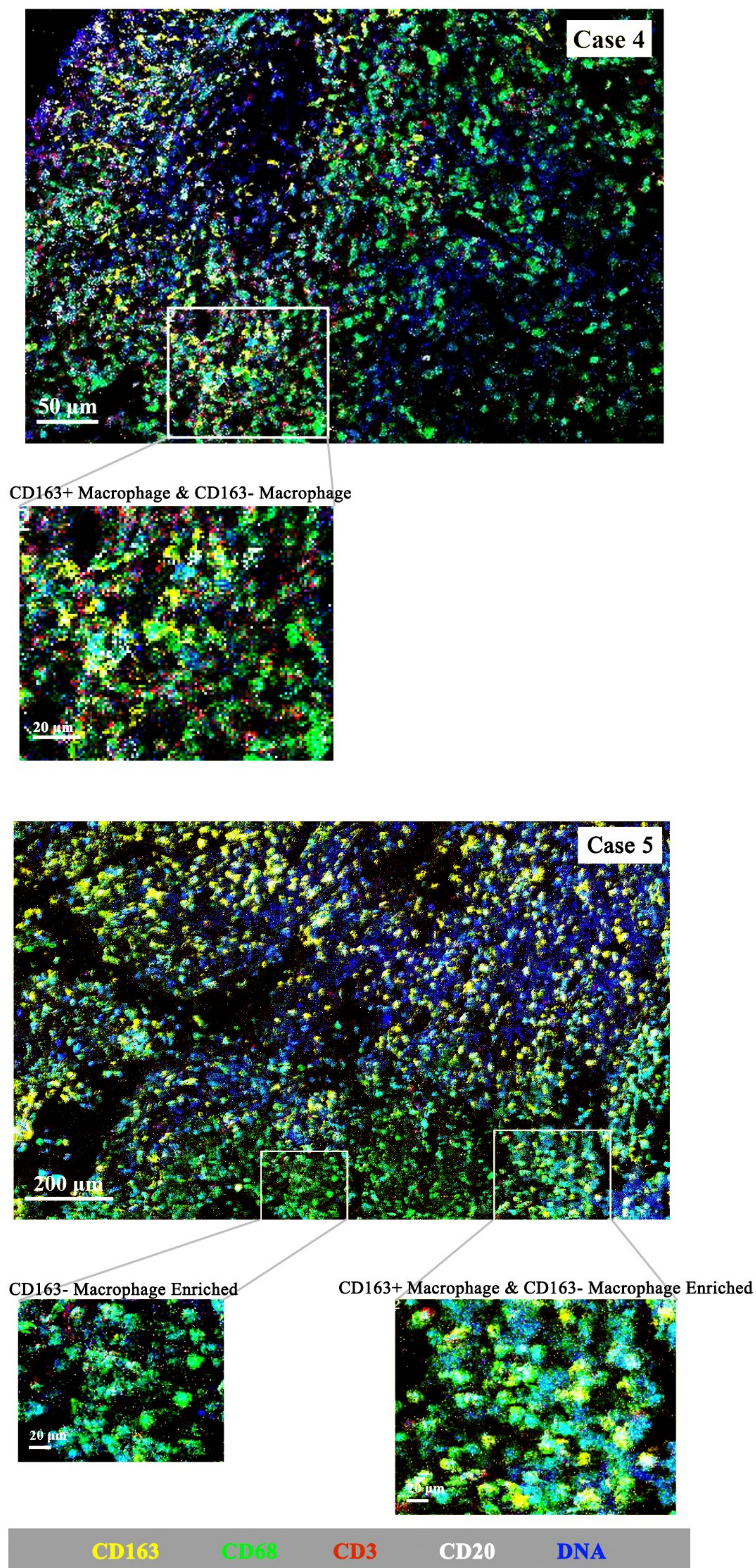

E

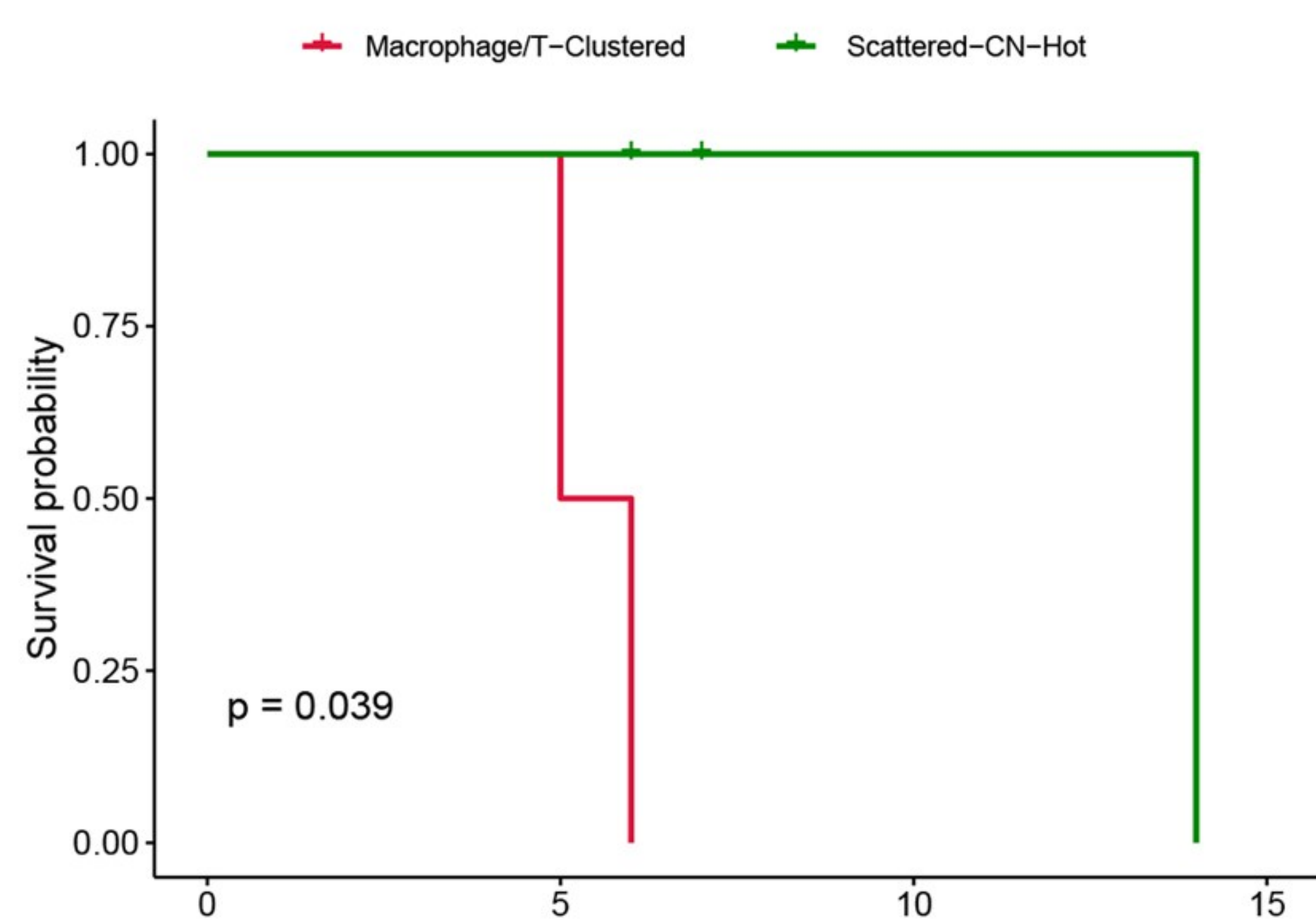

Figure S5

Supplement: Supplementary file 5 — Additional file 5: Figure S5. (A) Heatmap depicting the various immune CNs across the 5 cases with metastatic ccRCC. (B) The difference in CNs between the responder and non-responder groups. (C) Representative IMC images of non-responder (cases 4 and 5) showing the macrophage/T-clustered characteristics. (D) Representative IMC images of responders (cases 1, 2 and3) displaying the scattered-CN-hot characteristics. (E) Survival analysis between the responder and non-responder groups. [file 12967_2023_4336_MOESM5_ESM.pdf]
